# The cellular and molecular cardiac tissue responses in human inflammatory cardiomyopathies after SARS-CoV-2 infection and COVID-19 vaccination

**DOI:** 10.1038/s44161-025-00612-6

**Published:** 2025-02-24

**Authors:** Henrike Maatz, Eric L. Lindberg, Eleonora Adami, Natalia López-Anguita, Alvaro Perdomo-Sabogal, Lucía Cocera Ortega, Giannino Patone, Daniel Reichart, Anna Myronova, Sabine Schmidt, Ahmed Elsanhoury, Oliver Klein, Uwe Kühl, Emanuel Wyler, Markus Landthaler, Schayan Yousefian, Simon Haas, Florian Kurth, Sarah A. Teichmann, Gavin Y. Oudit, Hendrik Milting, Michela Noseda, Jonathan G. Seidman, Christine E. Seidman, Bettina Heidecker, Leif E. Sander, Birgit Sawitzki, Karin Klingel, Patrick Doeblin, Sebastian Kelle, Sophie Van Linthout, Norbert Hubner, Carsten Tschöpe

**Affiliations:** 1 Cardiovascular and Metabolic Sciences, Max Delbrück Center for Molecular Medicine in the Helmholtz Association (MDC), Berlin, Germany; 2 DZHK (German Centre for Cardiovascular Research), partner site Berlin, Berlin, Germany; 3 Department of Medicine I, University Hospital, LMU Munich, Munich, Germany; 4 Department of Genetics, Harvard Medical School, Boston, MA USA; 5 Cardiovascular Division, Brigham and Women’s Hospital Boston, Boston, MA USA; 6 Berlin Institute of Health (BIH) at Charité – Universitätsmedizin Berlin, BIH Center for Regenerative Therapies (BCRT), Berlin, Germany; 7 Berlin Institute for Medical Systems Biology (BIMSB), Max Delbrück Center for Molecular Medicine in the Helmholtz Association (MDC), Berlin, Germany; 8 Institut für Biologie, Humboldt Universität zu Berlin, Berlin, Germany; 9 Charité – Universitätsmedizin Berlin, Berlin, Germany; 10 German Cancer Consortium (DKTK), Partner Site Berlin, DKFZ and Charité – Universitätsmedizin Berlin, Berlin, Germany; 11 Berlin Institute of Health at Charité – Universitätsmedizin Berlin, Berlin, Germany; 12 Department of Infectious Diseases, Respiratory Medicine and Critical Care, Charité – Universitätsmedizin Berlin, corporate member of Freie Universität Berlin and Humboldt – Universität zu Berlin, Berlin, Germany; 13 Cellular Genetics Programme, Wellcome Sanger Institute, Wellcome Genome Campus, Hinxton, UK; 14 Cavendish Laboratory, Department of Physics, University of Cambridge, Cambridge, UK; 15 Division of Cardiology, Department of Medicine, Faculty of Medicine and Dentistry, University of Alberta, Edmonton, AB Canada; 16 Mazankowski Alberta Heart Institute, Faculty of Medicine and Dentistry, University of Alberta, Edmonton, AB Canada; 17 Erich and Hanna Klessmann Institute, Heart and Diabetes Center NRW, University Hospital of the Ruhr–University Bochum, Bad Oeynhausen, Germany; 18 National Heart and Lung Institute, Imperial College London, London, UK; 19 British Heart Foundation Centre for Research Excellence and Centre for Regenerative Medicine, Imperial College London, London, UK; 20 Howard Hughes Medical Institute, Chevy Chase, MD USA; 21 Department of Cardiology, Angiology and Intensive Medicine CBF, Deutsches Herzzentrum der Charité – Universitätsmedizin Berlin, Berlin, Germany; 22 Translational Immunology, Berlin Institute of Health at Charité – Universitätsmedizin Berlin, Berlin, Germany; 23 Cardiopathology, Institute for Pathology and Neuropathology, University Hospital Tübingen, Tübingen, Germany; 24 Department of Cardiology, Angiology and Intensive Care, Campus Virchow, Deutsches Herzzentrum der Charité – Universitätsmedizin Berlin, Berlin, Germany; 25 Helmholtz-Institute for Translational AngioCardioScience (HI-TAC) of the Max Delbrück Center for Molecular Medicine in the Helmholtz Association (MDC) at Heidelberg University, Heidelberg, Germany

**Keywords:** Transcriptomics, Cardiovascular diseases

## Abstract

Myocarditis, characterized by inflammatory cell infiltration, can have multiple etiologies, including severe acute respiratory syndrome coronavirus 2 (SARS-CoV-2) infection or, rarely, mRNA-based coronavirus disease 2019 (COVID-19) vaccination. The underlying cellular and molecular mechanisms remain poorly understood. In this study, we performed single-nucleus RNA sequencing on left ventricular endomyocardial biopsies from patients with myocarditis unrelated to COVID-19 (Non-COVID-19), after SARS-CoV-2 infection (Post-COVID-19) and after COVID-19 vaccination (Post-Vaccination). We identified distinct cytokine expression patterns, with interferon-γ playing a key role in Post-COVID-19, and upregulated *IL16* and *IL18* expression serving as a hallmark of Post-Vaccination myocarditis. Although myeloid responses were similar across all groups, the Post-Vaccination group showed a higher proportion of CD4^+^ T cells, and the Post-COVID-19 group exhibited an expansion of cytotoxic CD8^+^ T and natural killer cells. Endothelial cells showed gene expression changes indicative of vascular barrier dysfunction in the Post-COVID-19 group and ongoing angiogenesis across all groups. These findings highlight shared and distinct mechanisms driving myocarditis in patients with and without a history of SARS-CoV-2 infection or vaccination.

## Abstract (web summary)

Maatz, Lindberg et al. identify molecular alterations and immune response changes in endomyocardial biopsies from patients with myocarditis after COVID-19 infection, after anti-COVID-19 vaccination or from non-COVID-related causes.

## Main

Coronavirus disease 2019 (COVID-19) is primarily a respiratory disease, but systemic and cardiovascular involvement can occur, and acute cardiac injury^1,2^ with elevation of serum troponins is not uncommon after severe acute respiratory syndrome coronavirus 2 (SARS-CoV-2) infection. Strikingly, the risk and 1-year burden of cardiovascular diseases in survivors of acute COVID-19 are substantial, and the risk to specifically develop myocarditis is approximately fivefold increased^3^. In children/young adults, SARS-CoV-2 infection can lead to multisystem inflammatory syndrome (MIS-C), with myocarditis being the most prevalent clinical feature^4^. Cardiac injury can also rarely be induced after receipt of vaccines against COVID-19, in particular those based on mRNA technology^5,6^. Although evidence for direct SARS-CoV-2-mediated induction of myocardial injury is limited, the infection can elicit intense systemic release of cytokines, possibly leading to a secondary cardiac inflammatory response. Likewise, vaccine-associated myocardial inflammation and injury was shown to be characterized by systemic ‘cytokinopathy’, activated cytotoxic lymphocytes or induction of IL1-RA in the blood^7,8^. Using immunohistochemical staining of cardiac tissue from patients with clinically suspected myocarditis after SARS-CoV-2 infection or after COVID-19 mRNA vaccination, in particular a macrophage-dominated infiltrate was consistently observed^2,9^ that also was reported for SARS-CoV^10^. Furthermore, in patients with post-vaccination myocarditis, primarily CD4^+^ over CD8^+^ lymphocytic infiltrates were described^11^. However, the precise participating immune cell subsets and molecular changes driving their activation in cardiac tissue, as well as cardiac cell type resolved molecular responses, are incompletely characterized. Also, short-term clinical outcomes of cardiac inflammation are more favorable after vaccination, with most cases being mild. This raised the question of how the underlying cellular and molecular mechanisms compare in these different disease entities and whether they differ from pre-pandemic forms of myocarditis.

In the present study, we performed single-nucleus RNA sequencing (snRNA-seq) on cardiac tissue from symptomatic patients who clinically presented with pathological laboratory, electrocardiogram (ECG) and/or non-invasive imaging results of acute myocarditis and had undergone endomyocardial biopsies (EMBs) for diagnostic purposes. We studied EMBs from patients after SARS-CoV-2 infection (including two SARS-CoV-2-related patients with MIS-C) or after SARS-CoV-2 vaccination as well as from patients with histologically confirmed lymphocytic myocarditis that were mostly taken before the pandemic or had no signs of SARS-CoV-2 infection. Comparison of the different myocardial inflammatory modalities revealed common and divergent compositional cellular changes as well as pro-inflammatory and anti-inflammatory transcriptional signatures in patients with Non-COVID-19 myocarditis and those with myocarditis after SARS-CoV-2 infection or vaccination.

## Results

### Patient cohort and clinical presentation

**Fig. 1:**
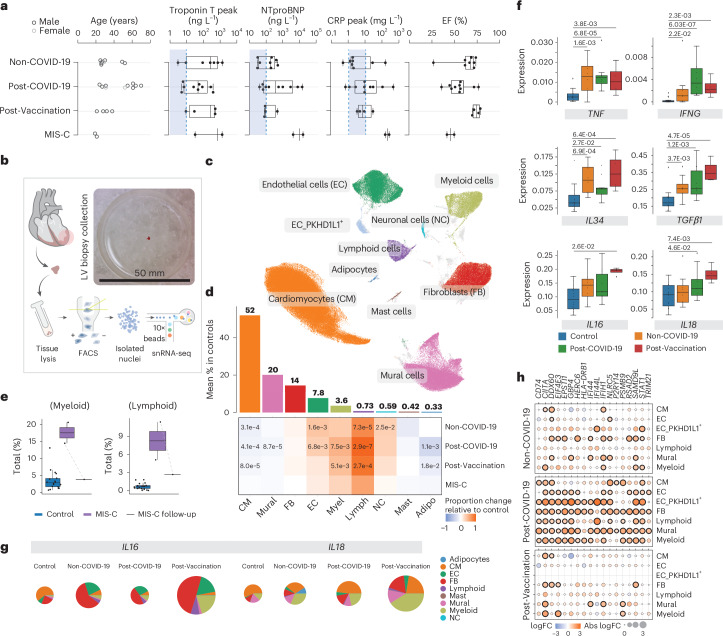
Distinct cellular and molecular signatures in Non-COVID-19, Post-COVID-19 and Post-Vaccination inflammatory cardiomyopathies. **a**, Infographic depicting metadata for Non-COVID-19 (*n* = 8), Post-COVID-19 (*n* = 10), Post-Vaccination (*n* = 4) and MIS-C (*n* = 2) patients. Left panel, patient age; women and men, light and dark gray, respectively. Middle panels, box plots showing serum levels of troponin T, NT-pro-BNP and CRP; dashed lines and blue areas indicate normal ranges. Right panel, box plots showing left ventricular ejection fraction (EF). **b**, snRNA-seq workflow schematic: EMBs were lysed and nuclei purified using FACS. Nuclei were processed using 10x Chromium 3′ chemistry. Image shows EMB size before nuclei isolation. **c**, UMAP embedding of 205,596 nuclei delineated 10 cardiac cell types and unassigned populations (gray). **d**, Upper panel, mean cell type abundances (%) in controls (*n* = 18). Lower panel, proportional changes of cell types in Non-COVID-19 (*n* = 8), Post-COVID-19 (*n* = 10) and Post-Vaccination (*n* = 4) versus control. Color scale: red (increase) and blue (decrease). Significant *P* values (FDR ≤ 0.05) are shown. *P* values were calculated with the two-sided *t*-test based on CLR-transformed values with Benjamini–Hochberg correction. MIS-C significance was not calculated due to low sample size (*n* = 2). **e**, Proportions of myeloid and lymphoid cells in control (*n* = 18) and MIS-C (*n* = 2) groups as well as in a follow-up biopsy from one patient with MIS-C.The patient with MIS-C with pre-treatment and post-treatment EMBs is indicated by a dashed line. Significance was not calculated due to low sample size (*n* = 2). **f**, Box plots as described in **a** showing snRNA-seq pseudo-bulk expression levels of cytokines in cardiac tissue from patient and control groups (control: *n* = 18, Non-COVID-19: *n* = 8, Post-COVID-19: *n* = 10, Post-Vaccination: *n* = 4). Significant *P* values (FDR ≤ 0.05) are shown. *P* values were calculated using the quasi-likelihood *F*-test with Benjamini–Hochberg correction. **g**, Pie charts comparing cell type resolved absolute mean expression levels of *IL16* and *IL18* between conditions. Pie size reflects absolute detection levels; colors indicate relative cell type contribution. **h**, Dot plots showing differential expression of IFNγ response genes in patient groups relative to controls in major cardiac cell types. Dot colors indicate log_2_-transformed FCs (log_*2*_FCs). Dot sizes indicate absolute log_2_FC. Black circles indicate significance (FDR ≤ 0.05). *P* value calculations are as in **f**. Box plots in **a**, **e** and **f**: boxes show interquartile range (IQR); vertical bars indicate the median; and whiskers extend from minimum to maximum values. Dots show individual measurements per patient. Adipo, adipocyte; Lymph, lymphoid; Myel, myeloid. Source data

Our clinical cohort consisted of (1) patients with ‘classical’ lymphocytic myocarditis (Non-COVID-19, *n* = 8); (2) patients with signs of acute myocarditis after SARS-CoV-2 infection (Post-COVID-19, *n* = 10); (3) patients with signs of acute myocarditis after vaccination against COVID-19 (Post-Vaccination, *n* = 4); (4) patients with MIS-C with signs of acute myocarditis (*n* = 2); and (5) control donor left ventricular (LV) tissue that we analyzed previously^12,13^. All patients presented with symptoms including chest pain, palpitations, fever, shortness of breath, malaise and/or general weakness and fatigue and an overall increase of cardiac damage-indicating biomarkers (troponin T, N-terminal prohormone of brain natriuretic peptide (NT-pro-BNP), creatine kinase or creatine kinase MB) and C-reactive protein (CRP) levels (Fig. 1a). ECG, echocardiography or signs of recent or ongoing myocardial damage in cardiac magnetic resonance imaging (MRI), ranging from normal or non-specific to borderline low or abnormal, are summarized in Extended Data Table 1. All patients underwent LV EMBs and left heart catheterization after routine non-invasive diagnostic workup, and angiography had failed to elucidate any other specific cause of heart failure, such as coronary artery disease. Post-COVID-19 patients and patients with MIS-C were previously tested positive for SARS-CoV2 infection by nasopharyngeal swab test polymerase chain reaction (PCR). Most Post-Vaccination patients experienced symptom onset within days after the second dose of the vaccine. Consistent with prior reports^7,9,14^, the cohort was predominantly male (87.5%; Non-COVID-19: 87.5%, Post-COVID-19: 80%, Post-Vaccination: 100% and MIS-C: 100%) with an average age of 37 ± 16 years (range, 19–70 years). The age of the two patients with MIS-C was 20 years and 21 years. Post-COVID-19 patients were slightly older than the other patients (Fig. 1a and Extended Data Table 1). Selection of Non-COVID-19 patients was based on positive EMB results showing lymphocytic myocarditis and similarities in sex and age compared to the other disease groups. In the MIS-C group, one patient underwent an additional EMB, 6 months after combined immunosuppression with prednisolone and azathioprine.

**Extended Data Fig. 1:**
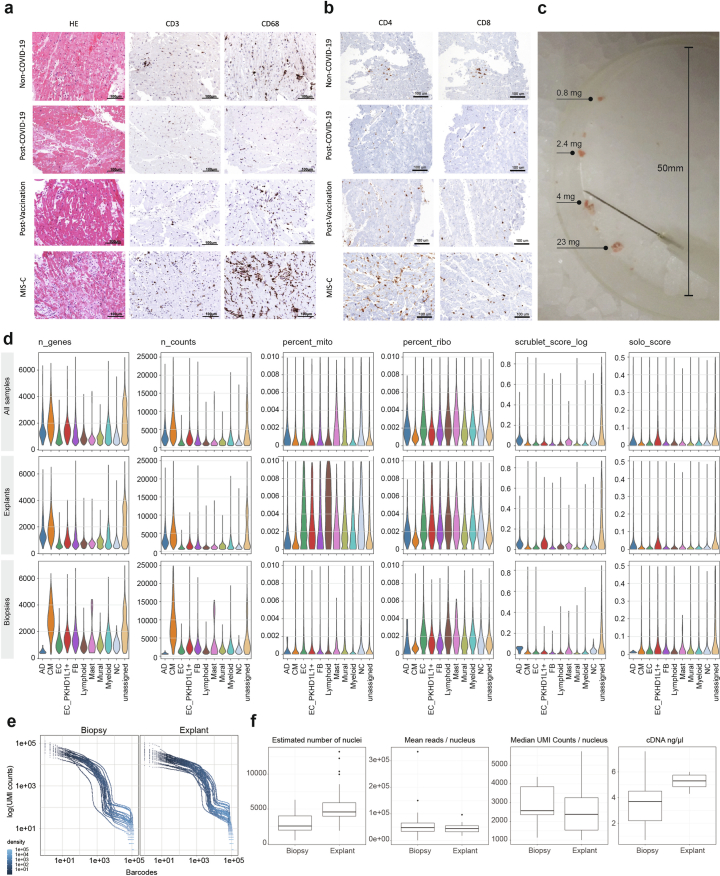
Immunohistochemistry and snRNAseq library quality metrics. **a)** Images of Hematoxylin eosin- (HE), CD3-, and CD68-stained and **b)** of CD4- and CD8- stained EMB sections. Representative images from each group are shown. Stainings on EMB sections were performed for all patients (Non-COVID-19: n = 8, Post-COVID-19: n = 10, Post-Vaccination: n = 4, MISC: n = 2). Cell counts are presented in Extended Data Table 1. **c)** Size of human left ventricular endomyocardial cardiac biopsies (EMBs) for snRNA-seq. The image shows different EMB tissue sizes and their weight before nuclei isolation in a 5 cm dish on ice. **d)** Violin plots showing number of detected genes (n_genes), detected UMIs (n_counts), fraction of UMIs mapping to mitochondrial-encoded genes and ribosomal genes and doublet probabilities according to scrublet and solo per nucleus within a cell-type. Distributions have been computed for all samples and subsetted for explants and EMB samples only. **e)** Barcode rank plots across all EMB (n = 25) and explant samples (controls n = 18). Clear distinction between nuclei containing droplets and empty droplets (background ambient RNA) indicated a low overall background. **f)** Box plots showing snRNA-seq library quality metrics. Boxes depict the interquartile range (IQR), horizontal bars represent the median, whiskers extend to 1.5 × IQR. Data points outside the whiskers show measurements beyond Q1 or Q3 ± 1.5* IQR. Estimated numbers of nuclei pre-QC filtering, mean reads per nucleus in the fastq files, median UMI counts per cell and the cDNA concentration per sample were depicted between EMB (Non-COVID-19, Post-COVID-19, Post-Vaccination, PIMS; n = 25) and explant tissue material (controls n = 18). Source data

Clinical histopathology and immunostaining on EMBs identified substantial widespread increased interstitial macrophage infiltration in all patients and, additionally, lymphocytic myocarditis in 30% of Post-COVID-19 patients, in 25% of Post-Vaccination patients and in 100% of patients with MIS-C (Extended Data Table 1 and Extended Data Fig. 1a,b). Our observations are in agreement with previous reports, where most post-COVID-19 and mRNA-vaccinated patients with signs of myocarditis showed predominantly macrophage infiltrates into the myocardium^2,9^. No SARS-CoV-2 genome was detected by PCR in EMBs of Post-COVID-19 patients and patients with MIS-C. EMBs that were not used for diagnostic workup were included for snRNA-seq analyses to investigate the cellular and molecular changes of myocardial inflammatory responses across the different disease entities.

### Myocarditis-associated changes in cardiac tissue composition

**Extended Data Fig. 2:**
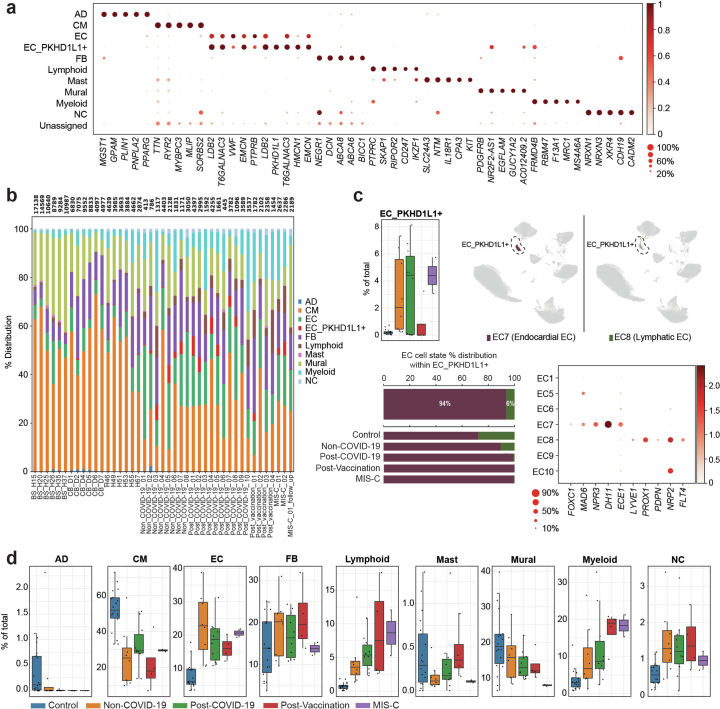
Cell-type composition of EMBs and control transmural cardiac tissues. **a)** Dot plot shows expression of the top five selected marker genes for each cardiac cell type. Dot size corresponds to fraction (%) of expressing nuclei; colour indicates scaled mean expression level. **b)** Stacked bar plots showing the cell type composition of each sample. The numbers above each bar show the total number of recovered nuclei. **c)** *Upper* l*eft panel:* Box plot showing PKHD1L1 + EC frequency in LV for patients and control groups (Control: n = 18, Non-COVID-19: n = 8, Post-COVID-19: n = 10, Post-Vaccination: n = 4, MISC: n = 2). Boxes depict the interquartile range (IQR), horizontal bars represent the median, whiskers extend to 1.5 × IQR, dots show the value of each patient. *Upper right panel:* UMAPs highlighting endocardial EC (EC7) or lymphatic EC (EC8) cell states within the EC_PKHD1L1+ cell population. All other cell types are shown in grey; Lower left *panel:* Stacked barplot showing EC state distribution (%) within the EC_PKHD1L1+ population (*top*) and splitted by condition (*bottom*); Lower *right panel:* Dot plot shows marker genes of EC7 and EC8 cell states. Dot size corresponds to fraction (%) of expressing nuclei; colour indicates mean expression level. **d)** Box plots showing cell type frequencies in LV per patient and control groups (Control: n = 18, Non-COVID-19: n = 8, Post-COVID-19: n = 10, Post-Vaccination: n = 4, MISC: n = 2). Boxes depict the interquartile range (IQR), horizontal bars represent the median, whiskers extend to 1.5 × IQR, dots show the value of each patient. *P*-values are shown in Fig. 1d. Source data

To generate snRNA-seq libraries, we isolated nuclei from EMBs using our previous protocol with modifications (Fig. 1b and Methods). For comparison to healthy hearts, we used reported full-thickness LV snRNA-seq data from 18 healthy controls^12,13^ (Extended Data Table 2). Despite the small amount of input material, the resulting EMB sequencing data were of similar quality to those of the full-thickness healthy heart samples (Extended Data Fig. 1c–f). After pre-processing and quality control filtering, nuclei were integrated using Harmony, followed by constructing manifolds using uniform manifold approximation and projections (UMAPs). In total, we analyzed 205,596 nuclei. Clustering identified 10 major cell types (Fig. 1c, Extended Data Fig. 2a,b and Supplementary Table 1) encompassing cardiomyocytes (CMs), fibroblasts (FBs), mural cells containing pericytes (PCs) and smooth muscle cells (SMCs), endothelial cells (ECs), PKHD1L1-expressing ECs (EC_PKHD1L1^+^) comprising mostly endocardial cells and few lymphatic ECs (Extended Data Fig. 2c), adipocytes, neural cells, mast cells and lymphoid and myeloid cells.

Using center log ratio (CLR)–transformed abundance of cell types^13^ (excluding EC_PKHD1L1^+^; see Methods), we compared the cellular composition of healthy versus patient hearts. In line with EMB immunohistochemistry results, myeloid cell proportions were increased in Post-COVID-19 and Post-Vaccination, whereas, in the Non-COVID-19 group, this increase did not reach statistical significance (Fig. 1d and Extended Data Fig. 2d). Surprisingly, we observed increased proportions of lymphoid cells for all patient groups (Fig. 1d) and, indeed, all Post-COVID-19 and Post-Vaccination patients (Extended Data Fig. 2b,d). This was in contrast to the clinical immunohistochemistry results where only a fraction of Post-COVID-19 and Post-Vaccination patients showed lymphocytic myocarditis, defined by the presence of CD3^+^ T cells. Similar to findings in end-stage heart failure^13^ and in hearts of deceased patients with COVID-19 (ref. ^15^), ECs were significantly expanded in Non-COVID-19 and Post-COVID-19. FB abundance remained unchanged, whereas CM and mural cell proportions were modestly reduced across patient groups.

Patients with MIS-C showed increased proportions of immune cells (lymphoids and myeloids), similar to the other patient groups (Fig. 1d and Extended Data Fig. 2d). Strikingly and as expected, comparing the cellular composition of the matching EMBs from one patient with MIS-C showed a marked reduction of immune cells after treatment, indicating normalization of immune cell numbers (Fig. 1e). Due to the low sample size (*n* = 2), throughout this paper we report MIS-C results only for differences in cell type and state abundances without statistical testing.

### Distinct cytokine and inflammasome expression signatures

**Extended Data Fig. 3:**
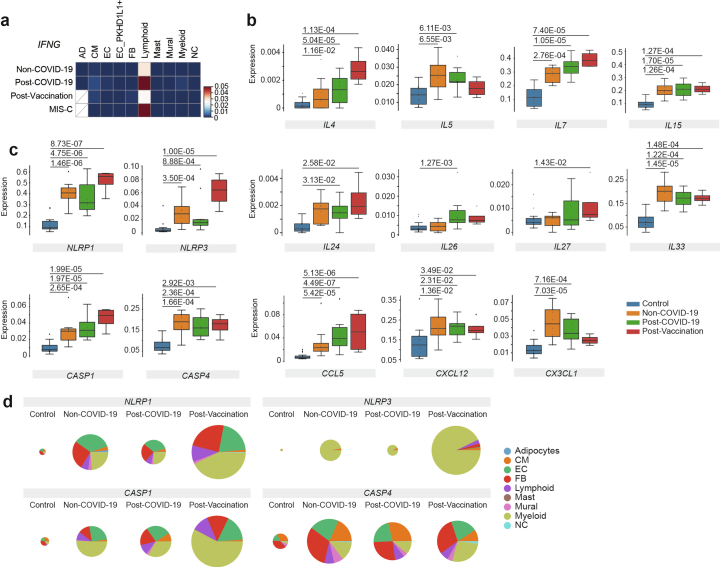
Elevated gene expression of pro-inflammatory markers. **a)** Matrix plot showing *IFNG* expression levels per cell-type. The Colour scale corresponds to the average expression level per cardiac cell-type. **b,c)** Box plots showing snRNA-seq expression levels (pseudo-bulk) in cardiac tissue from control (n = 18), Non-COVID-19 (n = 8), Post-COVID-19 ( = 10) and Post-Vaccination (n = 4) of (b) differentially expressed cytokines, chemokines and (c) components of the inflammasome complex. Boxes depict the interquartile range (IQR), horizontal bars represent the median, whiskers extend to 1.5 × IQR, dots show the value of each patient. P-values were calculated using the quasi-likelihood F-test and were adjusted for multiple testing (Benjamini Hochberg). **d)** Pie charts comparing mean absolute expression levels of inflammatory marker genes that are upregulated particularly in the Post-Vaccination group (*NLRP1, NLRP3, CASP1, CASP4)*. The pie size reflects absolute detection levels, colours indicate the relative contribution per cell type. Source data

We determined differentially expressed genes (DEGs) in cardiac tissue using a pseudo-bulk approach that aggregated all nuclei from the same individual (Supplementary Table 2) and detected increased expression of the pro-inflammatory cytokine genes *TNF* and *IFNG* across patient groups (Fig. 1f). *IFNG* was solely expressed in lymphocytes and especially elevated in the Post-COVID-19 and MIS-C groups (Fig. 1f and Extended Data Fig. 3a). Additional interleukin and leukocyte recruiting chemokine (C-X-C and C-C motif) ligand family member encoding genes were upregulated in the patient groups (Extended Data Fig. 3b), consistent with increased immune cell abundances.

We noted stronger upregulation of *IL34*, a macrophage growth and survival factor, in the Non-COVID-19 and Post-Vaccination groups than in the Post-COVID-19 group (Fig. 1f). In an inflammatory context, IL-34-derived macrophages suppress pro-inflammatory polarization of T cells^16^. Furthermore, *TGFB1* encoding immunosuppressive TGF-β1 was upregulated in all patient groups but highest in the Post-Vaccination group. *IL16* and *IL18* expression was uniquely increased in the Post-Vaccination group, and expression of inflammasome components such as pattern recognition receptors *NLRP1* and *NLRP3* and caspases *CASP1* and *CASP4* necessary for IL18 activation^17^ were highest in this group (Fig. 1f and Extended Data Fig. 3c). These genes were expressed in multiple cell types, but contribution of myeloids to their overall expression levels was greater in the Post-Vaccination group compared to all other patient groups (Fig. 1g and Extended Data Fig. 3d), suggesting a distinctive myeloid origin of this pathway in the Post-Vaccination group, consistent with previous findings^18^.

We then determined DEGs in pseudo-bulks of individual cell types and performed gene set enrichment analyses (GSEAs) on DEGs to identify disease-associated pathways. Strikingly, in the Post-COVID-19 group, upregulated gene sets related to interferon-γ (IFNγ) signaling in multiple cell types (Fig. 1h and Supplementary Table 3).

### Myeloid expansion and compositional changes in myocardits

**Fig. 2:**
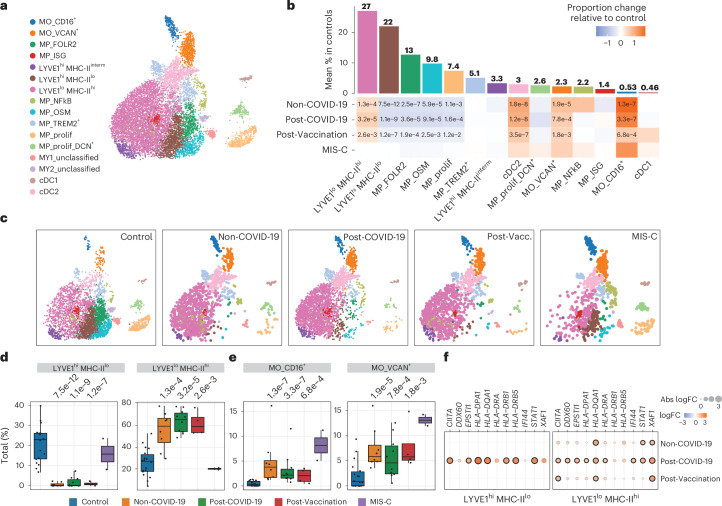
Myeloid compositional changes and gene expression alterations in cardiac inflammation. **a**, UMAP embedding delineated 16 myeloid cell states. **b**, Upper panel, mean abundance (%) of myeloid cell states in control left ventricles (*n* = 18). Lower panel, proportional changes of myeloid cell states in the four patient groups (Non-COVID-19: *n* = 8, Post-COVID-19: *n* = 10, Post-Vaccination: *n* = 4). Color scale: red (increase) and blue (decrease). *P* values are indicated for significant proportional changes (FDR ≤ 0.05). *P* values were calculated using the two-sided *t*-test based on CLR-transformed values with Benjamini–Hochberg correction. For MIS-C, significance was not calculated due to low sample size (*n* = 2). **c**, Condition-split UMAP showing compositional shifts in myeloid cell states across patient groups and controls. **d**,**e**, Box plots showing distribution of Lyve1^hi^MHCII^lo^ and Lyve1^lo^MHCII^hi^ (**d**) and CD16^+^ and VCAN^+^ monocytes (**e**) across control and disease groups (control: *n* = 18, Non-COVID-19: *n* = 8, Post-COVID-19: *n* = 10, Post-Vaccination: *n* = 4). Boxes depict the interquartile range (IQR); horizontal bars indicate the median; whiskers extend to 1.5× IQR; and dots show the value of each patient. *P* values are indicated for significant proportional changes, FDR < 0.05. *P* value calculations are as described in **b**. **f**, Dot plots showing differential expression of selected IFNγ response and MHC-II genes in patient groups relative to control across Lyve1^hi^MHCII^lo^ and Lyve1^lo^MHCII^hi^ (control: *n* = 18, Non-COVID-19: *n* = 8, Post-COVID-19: *n* = 10, Post-Vaccination: *n* = 4). Dot colors indicate log_2_-transformed FCs (log_2_FCs). Dot sizes indicate absolute log_2_FC. Black circles indicate significance (FDR ≤ 0.05). *P* values were calculated using the quasi-likelihood *F*-test and were adjusted for multiple testing (Benjamini–Hochberg). Genes are ordered alphabetically. Source data

**Extended Data Fig. 4:**
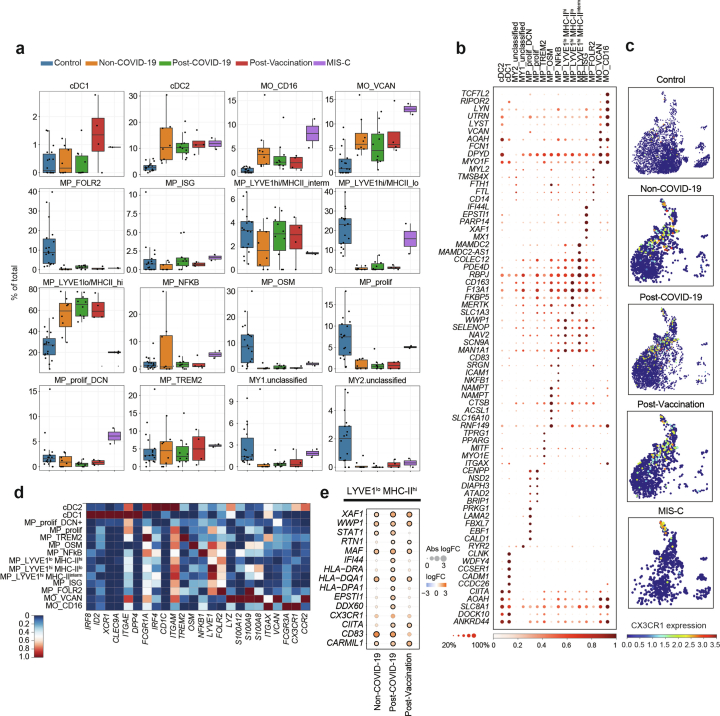
The cellular and molecular signatures of myeloid cells in cardiac inflammation. **a)** Box plots showing myeloid state frequencies in LV per patient in Non-COVID-19 (n = 8), Post-COVID-19 (n = 10), Post-Vaccination (n = 4), MIS-C (n = 2), and control (n = 18) groups. Boxes depict the interquartile range (IQR), horizontal bars represent the median, whiskers extend to 1.5 × IQR, dots show the value of each patient. *P*-values are shown in Fig. 2b. **b)** Dot plot showing the expression of the top five marker genes for each myeloid cell state. Dot size corresponds to fraction (%) of expressing nuclei; colour indicates scaled mean expression levels. **c)** Condition-split UMAP showing *CX3CR1* expression per myeloid nuclei across control and myocarditis groups. **d)** Matrix plot showing selected gene expression levels per myeloid state. Color scale corresponds to the scaled average expression levels. **e)** Dot plots showing differential expression of genes identified to be upregulated in Lyve1loMHCIIhi MP in patient groups relative to control. Dot colours indicate log2-transformed fold changes (log2FC). Dot sizes indicate absolute log2FC. Black circles indicate significance (FDR ≤ 0.05). Genes are ordered alphabetically. Source data

Characterization of myeloid cells identified nine previously described^13^ macrophage states, VCAN^+^ and non-classical CD16^+^ monocytes, two conventional dendritic cell states and one decorin (*DCN*)-expressing proliferative macrophage state (Fig. 2a,b, Extended Data Fig. 4a,b and Supplementary Table 4). Despite the varying extent of the overall myeloid expansion (Extended Data Fig. 2d), we observed similar striking compositional alterations in the myeloid populations in Non-Covid-19, Post-COVID-19 and Post-Vaccination groups compared to control (Fig. 2b and Extended Data Fig. 4a). Among pre-existing resident macrophages, LYVE1^hi^MHCII^lo^ with pivotal cardiac repair function^19^ was barely detectable, whereas proportions of LYVE1^lo^MHCII^hi^ that effectively stimulate T cell responses^19,20^ were strongly increased in these three patient groups (Fig. 2b–d). Furthermore, proportions of T cell–stimulating cDC2 were increased, whereas FB-interacting MP_OSM macrophages and MP_FOLR2 were decreased (Fig. 2b and Extended Data Fig. 4a). Reduced proliferating macrophage and expanded CD16^+^ and VCAN^+^ monocyte proportions (Fig. 2e and Extended Data Fig. 4a), as well as *CX3CR1* expression (circulating monocyte marker) (Extended Data Fig. 4c), indicated recruited monocytes as a source for the LYVE1^lo^MHCII^hi^ MP increase. Of note, VCAN^+^ monocytes expressed *CCR2* and pro-inflammatory mediators (*S100A12*, *S100A9* and *S100A8*) (Extended Data Fig. 4d). In a myocarditis mouse model, silencing of *CCR2* prevented cardiac monocyte accumulation and chronic decline of LV function^21^. Patients with MIS-C showed different compositional alterations of the myeloid cell population compared to the other patient groups, with no change in pre-existing resident macrophage proportions but an increase in monocyte proportions exceeding, by far, those observed in the other groups (Fig. 2e). Furthermore, *DCN*-expressing proliferative macrophages were enriched only in patients with MIS-C. Expression of FB genes in macrophages may indicate the acquisition of a fibrogenic phenotype^22,23^.

DEG analysis comparing myeloid states in patient groups versus control revealed the highest number of upregulated genes in LYVE1^lo^MHCII^hi^ macrophages (Supplementary Table 5), including *CARMIL1*, *MAF*, *WWP1* and *CD83*, indicating higher activation and interstitial migration capacities^24,25^ (Extended Data Fig. 4e). Contrary to reports for macrophages from lung of patients with severe COVID-19 and from blood of patients with perimyocarditis after vaccination^7,26^, we did not observe pro-fibrotic gene expression responses in cardiac myeloids. However, GSEA on DEGs per cell state identified upregulated gene sets related to IFNγ signaling in LYVE1^hi^MHCII^lo^, LYVE1^lo^MHCII^hi^ and proliferating macrophages as well as in cDC2 from Post-COVID-19 patients (Fig. 2f and Supplementary Table 3). IFNγ is known to increase pro-inflammatory activity in macrophages^27^ and to induce major histocompatibility complex class II (MHC-II) gene expression^28^. Indeed, macrophages in the Post-COVID-19 group showed upregulated class II MHC transactivator encoding *CIITA* and MHC-II genes (Fig. 2f).

### Distinct lymphocyte responses in Post-COVID-19 and Post-Vaccination

**Fig. 3:**
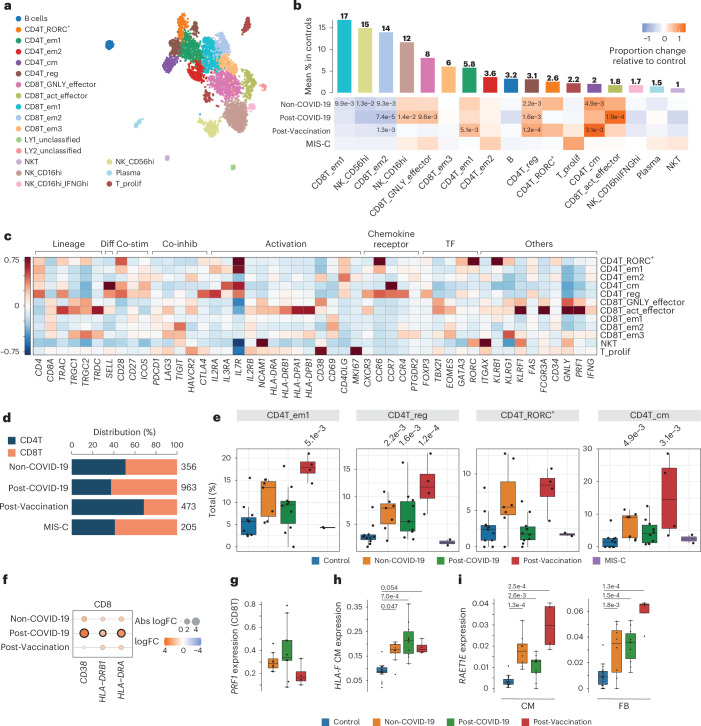
Distinct lymphocyte responses in cardiac tissue of Post-COVID-19 and Post-Vaccination patients. **a**, UMAP embedding of lymphoid cells delineated 19 lymphoid cell states. **b**, Upper panel, mean abundance (%) of lymphoid cell states in control left ventricles (*n* = 11). Lower panel, proportional changes of lymphoid cell states in Non-COVID-19 (*n* = 7), Post-COVID-19 (*n* = 9) and Post-Vaccination (*n* = 4). Color scale: red (increase) and blue (decrease). *P* values are indicated for significant proportional changes (FDR ≤ 0.05). *P* values were calculated using the two-sided *t*-test based on CLR-transformed values with Benjamini–Hochberg correction. For MIS-C, significance was not calculated due to low sample size (*n* = 2). Only samples with lymphoid counts greater than 30 were considered (Methods). **c**, Heatmap showing the z-score standardized expression of select marker genes (columns) per T cell cluster (rows). TF, transcription factor. **d**, Stacked bar chart indicating CD8T/CD4T ratios across conditions. *n* numbers are as described in **b**. **e**, Box plots showing distribution of CD4^+^ effector memory 1 (CD4T_em1), regulatory (CD4T_reg), RORC-expressing (CD4T_RORC^+^) and central memory (CD4T_cm) T cells. Significant *P* values (FDR ≤ 0.05) are shown. *n* numbers and *P* value calculations are as described in **b**. **f**, Dot plot showing differential expression of activation markers in pseudo-bulk CD8T cells across patient groups relative to control. Dot colors indicate log_2_-transformed FCs (log_2_FCs). Dot sizes indicate absolute log_2_FC. Black circles indicate significance (FDR ≤ 0.05). *P* values were calculated using the quasi-likelihood *F*-test with Benjamini–Hochberg correction. **g**, Box plots showing *PRF1* snRNA-seq expression levels in pseudo-bulk CD8T cells from patient groups (Non-COVID-19: *n* = 7, Post-COVID-19: *n* = 10, Post-Vaccination: *n* = 4; see Methods, DEG analysis). *P* value calculations are as described in **f**. *P* values were FDR > 0.05. **h**,**i**, Box plots showing snRNA-seq pseudo-bulk expression levels of *HLA-F* in CMs (**h**) and of *RAET1E* in CMs (**i**) (left) and FBs (right) from patient and control groups (control: *n* = 18, Non-COVID-19: *n* = 8, Post-COVID-19: *n* = 10, Post-Vaccination: *n* = 4). Significant *P* values (FDR ≤ 0.05) are shown. *P* values were calculated as described in **g**. Box plots in **e**, **g** and **h**: Boxes show interquartile range (IQR); vertical bars indicate the median; and whiskers extend from minimum to maximum values. Dots show individual measurements per patient. Source data

**Extended Data Fig. 5:**
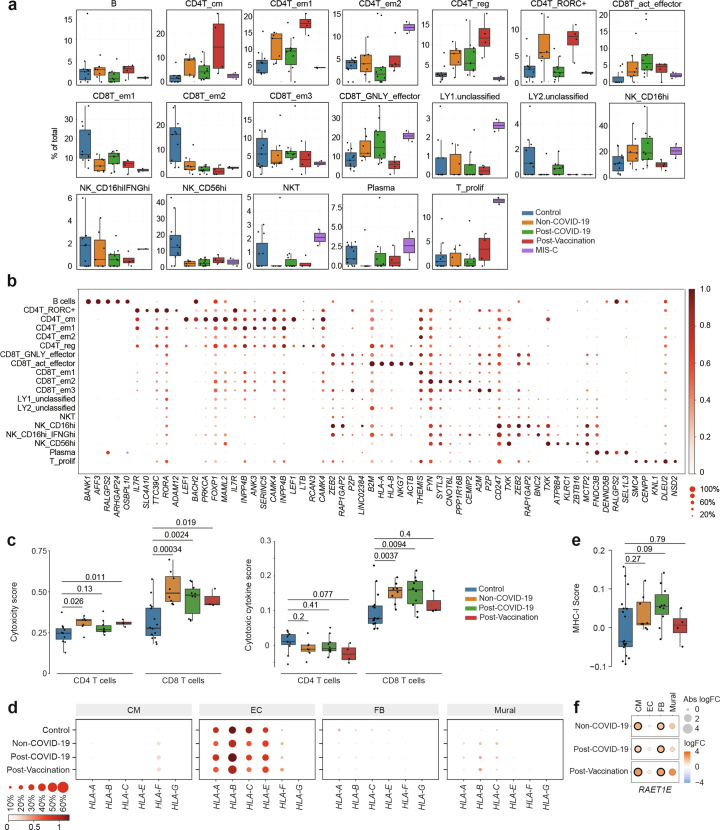
The cellular and molecular signatures of lymphoid cells in cardiac inflammation. **a)** Box plots showing lymphoid state frequencies in LV per patient in Non-COVID-19 ( = 8), Post-COVID-19 (n = 10), Post-Vaccination (n = 4), MIS-C (n = 2), and control (n = 18) groups. Boxes depict the interquartile range (IQR), horizontal bars represent the median, whiskers extend to 1.5 × IQR, dots show the value of each patient. *P*-values are shown in Fig. 4b. **b)** Dot plot showing the expression of the top five marker genes for each lymphoid cell state. Dot size corresponds to fraction (%) of expressing nuclei; colour indicates scaled mean expression levels. **c)** Cytotoxicity (right) or cytotoxic cytokines cores (left) across aggregated CD4+ or CD8 + T cells (control: n = 11; Non-COVID-19: n = 7; Post-COVID-19: n = 9; Post-Vaccination: n = 4). Scores were calculated across ‘cytotoxicity’^37^ and ‘cytotoxic cytokine’^38^ gene sets, respectively. Boxes depict the interquartile range (IQR), horizontal bars represent the median, whiskers extend to 1.5 × IQR, dots show the value of each patient. P-values were calculated using the two-sided Wilcoxen rank-sum test and were adjusted for multiple testing (Bonferoni correction). **d)** Dot plots showing expression of MHC-I genes across conditions in CM, EC, FB and mural cells. **e)** MHC-I gene expression score across aggregated non-immune cardiac cells shown as described in c); control: n = 18, Non-COVID-19: n = 8, Post-COVID-19: n = 10, Post-Vaccination: n = 4. Scores were calculated across all MHC-I genes. P-value calculations as described in c). **f)** Dot plots as described in b) showing differential expression of *RAET1E* in CM, EC, FB and murals cells in patient groups relative to control. Source data

Subcluster analyses of lymphoid cells revealed 10 T cell and three natural killer (NK) cell states as well as plasma, B cells and a small population of IFNγ-producing NK cells (Fig. 3a–c, Extended Data Fig. 5a,b and Supplementary Tables 6 and 7). Within the T cell population, we observed higher CD4^+^ to CD8^+^ T cell ratios in Non-COVID-19, and this effect was even more pronounced in Post-Vaccination, aligning with previously reported predominance of cardiac CD4^+^ lymphocytic infiltrates in response to COVID-19 vaccination^11^. In contrast, CD8^+^ T cells dominated in the Post-COVID-19 and MIS-C groups (Fig. 3d). Overall, CD8^+^ T cells displayed higher expression of gene sets related to cytotoxicity compared to CD4^+^ T cells (Extended Data Fig. 5c). The differences in T cell ratios between disease groups were attributable especially to a relative increase in regulatory, Tem1 and central memory CD4^+^ T cells in Non-COVID-19 and Post-Vaccination (Fig. 3b,e). Additionally, RORC^+^CD4^+^ T cells were increased in these groups, albeit not statistically significantly. Conversely, cytotoxic *GNLY*-expressing effector cell proportions and mature NK cells (NK_CD16^hi^) increased in Non-COVID-19, Post-COVID-19 and MIS-C but not in Post-Vaccination (Fig. 3b). Proliferating T cells were increased only in MIS-C (Extended Data Fig. 5a). CD8^+^ T cells in Post-COVID-19 furthermore showed upregulated expression of activation markers such as CD38 and HLA-DR (Fig. 3f) and higher *PRF1* expression levels encoding the cytotoxic effector molecule perforin than Non-COVID-19 and Post-Vaccination (Fig. 3g), suggesting an increased cytotoxic potential of CD8^+^ T cells in hearts of Post-COVID-19 patients. Interestingly, we identified highly activated CD16^+^CD8^+^ T cells (CD8T_act_effector) with strong cytotoxic functions and high similarity to CD16^+^CD8^+^ T cells previously identified in the blood of patients with severe COVID-19 (ref. ^29^). These cells were significantly enriched in cardiac tissue of Post-COVID-19 patients (Fig. 3b). Nuclei within this cluster expressed TRAC, TRDC and TRGC1/2, indicating a mixed cluster of αβ and γδ T cells that could not be separated further.

We explored our snRNA-seq data for transcriptional signatures that may suggest an impact of cytotoxic lymphocytes on non-immune cardiac cells. CD8^+^ T cells recognize and kill cells presenting perceived non-self-antigens via MHC-I molecules. Among cardiac non-immune cells, ECs showed the highest human leukocyte antigen (HLA) class I gene expression, whereas control CMs displayed barely detectable levels, consistent with previous findings^30,31^ (Extended Data Fig. 5d). Interestingly, in myocarditis, it was reported that MHC-I is upregulated on CMs and interstitial cells^30^. In our study, aggregated pseudo-bulk data of non-immune cardiac cells showed elevated, albeit not significant, HLA class I gene expression in disease conditions with higher levels in Post-COVID-19 (Extended Data Fig. 5e). In CMs, *HLA-F* was specifically upregulated across Non-COVID-19, Post-COVID-19 and Post-Vaccination conditions (Fig. 3h and Extended Data Fig. 5d). Additionally, in CMs and FBs, the MHC class I–like molecule RAET1E was particularly upregulated in Post-Vaccination (Fig. 3i and Extended Data Fig. 5f). RAET1E binds and activates NKG2D-expressing NK and CD8^+^ T cells, with RAET family members typically absent on normal cells but overexpressed under stress. This overexpression was reported to contribute to the development of autoimmunity^32^ and may be linked to an exacerbated inflammatory response.

### Elevated HIF1A and VEGFA expression in CMs

**Fig. 4:**
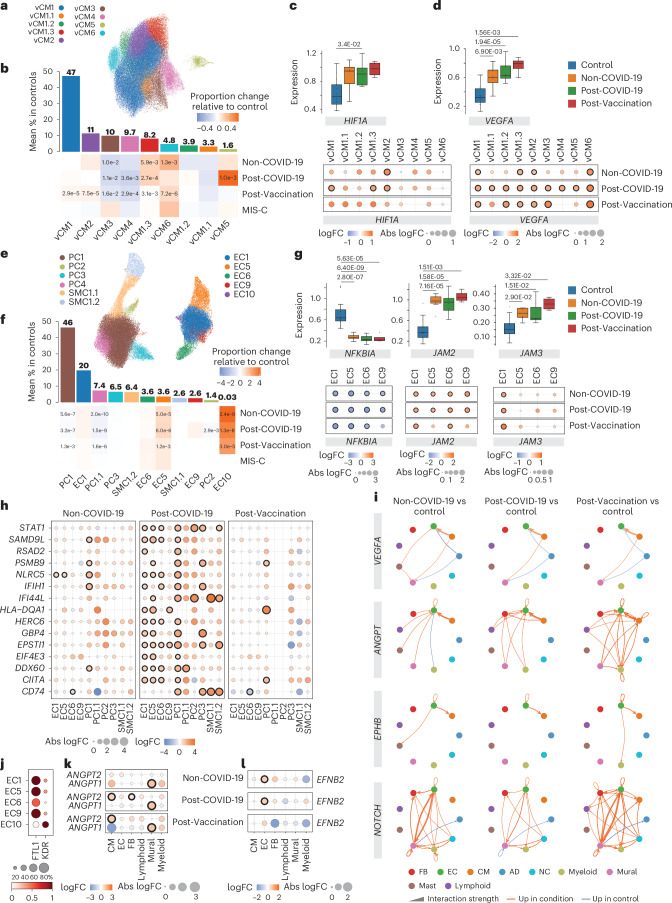
Vascular cells exhibit pro-angiogenic, inflammation and immune cell recruitment signatures across disease groups. **a**, UMAP embedding delineated nine CM states. **b**, Upper panel, mean CM state abundances (%) in controls (*n* = 18). Lower panel, proportional changes of CM states in Non-COVID-19 (*n* = 8), Post-COVID-19 (*n* = 10) and Post-Vaccination (*n* = 4) versus control. Color scale: red (increase) and blue (decrease). Significant *P* values (FDR ≤ 0.05) are shown. *P* values were calculated with the two-sided *t*-test based on CLR-transformed values with Benjamini–Hochberg correction. MIS-C significance was not calculated due to low sample size (*n* = 2). **c**, Upper panel, box plots showing *HIF1A* snRNA-seq pseudo-bulk expression levels in CMs. Boxes depict the interquartile range (IQR); horizontal bars indicte the median; whiskers extend to 1.5× IQR; and dots show the value of each patient. Significant *P* values (FDR ≤ 0.05) are shown. Lower panel, dot plots showing differential expression of *HIF1A* in patient groups relative to control across CM states. Dot colors indicate log_2_-transformed FCs (log_2_FCs). Dot sizes indicate absolute log_2_FC. Black circles indicate significance (FDR ≤ 0.05). Upper and lower panel, control: *n* = 18, Non-COVID-19: *n* = 8, Post-COVID-19: *n* = 10, Post-Vaccination: *n* = 4. *P* values were calculated using the quasi-likelihood *F*-test with Benjamini–Hochberg correction. **d**, *VEGFA* expression in CMs, shown as described in **c**. **e**, UMAP embedding delineated 11 vascular cell states. **f**, Vascular cell state abundances shown as described in **b**. **g**, *NFKBIA*, *JAM2* and *JAM3* expression in ECs, shown as described in **c**. **h**, Dot plots showing differential expression of IFNγ response genes in patient groups relative to control across vascular cell states. Dot plots, *n* numbers and *P* value calculations are as described in **c**. **i**, Circle plots showing significant cell–cell communication differences for the indicated pathways (*P* ≤ 0.05) between patient groups and controls. *P* values were computed from the one-sided permutation test with Bonferoni correction. Line thickness reflects interaction strength of sending and receiving cells; color indicates changes (orange: increased; blue: decreased); and arrows show signal directionality. **j**, Dot plot showing *FLT1* and *KDR* expression across EC states. Dot size corresponds to fraction (%) of expressing nuclei; color indicates scaled mean expression levels. **k**,**l**, *ANGPT1* and *ANGPT2* (**k**) and *EFNB2* (**l**) expression in cell types as described in **c**. AD, adipocyte; NC, neural cell. Source data

**Extended Data Fig. 6:**
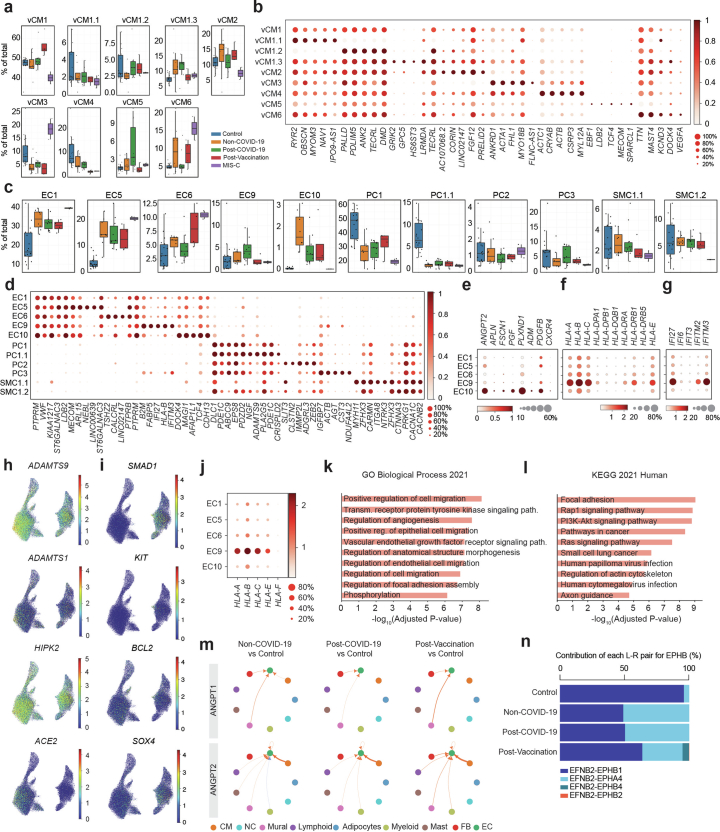
The cellular and molecular signatures in cardiomyocytes and vascular cells in cardiac inflammation. **a)** Box plots showing CM state frequencies in LV per patient in Non-COVID-19 ( = 8), Post-COVID-19 (n = 10), Post-Vaccination (n = 4), MIS-C (n = 2), and control (n = 18) groups. Boxes depict the interquartile range (IQR), horizontal bars represent the median, whiskers extend to 1.5 × IQR, dots show the value of each patient. *P*-values are shown in Fig. 5b. **b)** Dot plot showing the expression of the top five marker genes for each CM state. Dot size corresponds to fraction (%) of expressing nuclei; colour indicates scaled mean expression levels. **c)** Box plots showing EC state frequencies as described in a). *P*-values are shown in Fig. 5f. **d)** Dot plot as described in b) showing the expression of the top five marker genes for each EC and mural cell state. **e-g)** Dot plots as described in a) showing the expression of e) selected EC10-enriched genes f) MHC-I/-II genes and g) INF response genes across EC states. **h, i)** UMAP showing expression levels of selected genes per vascular cell nuclei across control and patient groups. **j)** Dot plots as described in a) showing the expression of MHC-I genes across EC states. **k, l)** Bar plots showing significant gene sets for EC10 from the k) GO Biological Process 2021 and l) KEGG 2021 Human gene lists. -log10 of the FDR is shown on the x-axis. P-values were calculated using GSEAs Fisher exact test. P-values were adjusted for multiple-testing using the Benjamini-Hochberg correction. **m)** Circle plot representations of significantly differential interactions of cell-cell communication for the ANGPT1 and ANGPT2 pathways (adjusted P-value ≤ 0.05) in patient groups compared to control. The line thickness represents the interaction strength of signals from sending and receiving cells. Colour is scaled from zero to maximum in disease versus controls (orange, increased; blue, decreased). The directionality of the signal is indicated with arrows. P-values were computed from the one-sided permutation test. **n)** Stacked bar plots showing the relative contribution of ephrin signalling ligand-receptor (L-R) pairs to the EPHB net signalling related to Fig. 4i in patient and control groups. Source data

Previous in vitro studies reported specific gene expression signatures after infecting induced pluripotent stem cell (iPSC)-derived CMs with SARS-CoV-2 (ref. ^33^). We did not observe a matching transcriptional response in our snRNA-seq CM data, neither in Post-COVID-19 patients nor in any other of the patient groups. Subclustering of the CM population identified previously reported cell states of the left ventricle^13^ (Fig. 4a,b, Extended Data Fig. 6a,b and Supplementary Tables 8 and 9) and one additional CM state (vCM6) that showed enrichment of genes related to regulation of heart rate by cardiac conduction (including, for example, *CAMK2D*, *KCND3*, *CACNA2D1*, *CTNNA3* and *CACNA1C*). vCM6 proportions were more abundant in all patient groups, especially in Non-COVID-19 and Post-Vaccination (Fig. 4b and Extended Data Fig. 6a), whereas vCM5 proportions were increased only in Post-COVID-19. vCM5 was described to participate in the cardiac conduction system^12^. Increased risks of cardiac arrhythmias were observed after a positive SARS-CoV-2 test^3,34^.

In CMs, we observed a significant upregulation of *HIF1A* in all patient groups compared to controls (Fig. 4c). *HIF1A* encodes the O_2_-regulated HIF1 subunit, and its expression can also be induced by various cytokines^35^. Local oxygen concentration in inflamed areas tends to decrease^36^. HIF1 functions as a master regulator of cellular and systemic homeostatic response to hypoxia, such as angiogenesis and vascular remodeling^37^. One notable HIF1A downstream target is the vascular endothelial growth factor A (*VEGFA*). Its product, VEGFA, is a powerful inducer of angiogenesis but can also induce vascular inflammation and increase vascular permeability^38^. *VEGFA* expression was upregulated in the overall CM population and across CM states in all patient groups (Fig. 4d).

### Pro-angiogenic and inflammatory gene expression in vascular cells

Subclustering of vascular cells (ECs and mural cells) delineated previously characterized capillary (EC1), arterial (EC5) and venous (EC6) ECs as well as two SMC states (SMC1.1 and SMC1.2) and three PC states (PC1–3)^13^ (Fig. 4e,f, Extended Data Fig. 6c–i and Supplementary Tables 10 and 11). We identified one additional PC state (PC1.1) with enriched expression of genes with anti-angiogenic capacity, including *ADAMTS9*, *ADAMTS1* and *HIPK2*, and two additional EC states, EC9 and EC10. EC9 was characterized by the expression of interferon-stimulated genes (ISGs). EC10 showed enriched expression of pro-angiogenic stem cell receptor *KIT*, *SMAD1* and tip cell marker genes (including *ANGPT2*, *PGF* and *PDGFB*) (Extended Data Fig. 6e), similar to ECs previously described to promote cardiac repair after myocardial infarction^39^. We observed across all patient groups decreased canonical PC1 and anti-angiogenic PC1.1 proportions. In contrast, EC5 (arterial) proportions were strongly increased, and EC1 (capillary) and EC6 (venous) proportions were barely increased. This indicated altered vascular cell ratios in all patient groups. ISG-expressing EC9 cells were increased in Post-COVID-19. These cells showed the highest HLA class I gene expression among EC states (Extended Data Fig. 6j), suggesting that they may represent a preferential target for cytotoxic CD8^+^ T cells within the EC population. EC10 cells were almost exclusively found in the patient groups except in MIS-C (Fig. 4f and Extended Data Fig. 6c).

DEG analysis identified consistently decreased *NFKBIA* expression in ECs within all patient groups (Fig. 4g). *NFKBIA* encodes the IκBα repressor that counteracts the pro-inflammatory transcription factor NF-κB. We furthermore noted a consistent upregulation of the junctional adhesion molecules encoding genes *JAM2* and *JAM3* in ECs across all patient groups (Fig. 4g). JAM2 and JAM3 are known to facilitate the migration and extravasation of immune cells through the endothelium^40^. We then performed GSEA on DEGs per vascular cell state and observed upregulated gene sets related to IFNγ signaling in EC1, EC5 and EC6 as well as PC1 and PC3 from Post-COVID-19 patients (Fig. 4h).

### Elevated angiogenesis-associated intercellular communication

By examining the expression of genes encoding for receptors and ligands, we inferred cell–cell communication using CellChat and detected increased VEGFA signaling from CMs to ECs in all patient groups (Fig. 4i). VEGFA promotes angiogenesis and vascular permeability primarily through its receptor VEGFR2 (KDR). Alternatively, it can bind to its decoy receptor VEGFR1 (FLT1), thus fine-tuning the angiogenic process and ensuring vascular quiescence and stability^41^.

Compared to other EC states, *KDR* showed highest and *FLT1* lower expression in EC10 (Fig. 4j), suggesting increased responsiveness to VEGFA. EC activation by VEGFA leads to formation of tip cell filopodia and facilitates migration, proliferation and survival^42^. Gene Ontology (GO) term analysis on genes expressed in EC10 indeed resulted in the identification of related terms (Extended Data Fig. 6k,l).

CellChat analyses furthermore identified increased angiogenesis-related angiopoietin (ANGPT), ephrin B (EPHB) and NOTCH signaling in all patient groups (Fig. 4i). Angiopoetin ligand ANGPT1 stabilizes vessels, and ANGPT2 is a VEGFA-dependent modulator of capillary structures and EC survival^43^. CellChat analyses predicted enhanced signaling of ANGPT1 from CMs and of ANGPT2 from mural cells to ECs (Extended Data Fig. 6m). *ANGPT1* and *ANGPT2* expression was increased in CMs and mural cells, respectively, across all patient groups (Fig. 4k). Ephrin B ligands and their Eph receptors play important roles in vessel growth and stabilization^44^. Expression of *EFNB2*, encoding a EPHB signaling ligand, was upregulated in ECs (Fig. 4l), and CellChat analyses predicted induction of autocrine signaling in ECs and increased signaling to CMs, where EFNB2 signaling was shown to play a protective role^45^. Furthermore, predicted EFNB2–EPHB1 ligand–receptor interactions identified in controls were shifted to EFNB2–EPHA4 in the patient groups (Extended Data Fig. 6n). Activation of EPHA4 in ECs was shown to increase monocyte adhesion^46^ and limit arteriogenesis through attenuated ANGPT2/Tie2 signaling^47^. NOTCH signaling plays a crucial role in regulating smooth muscle differentiation and blood vessel formation. In addition to increased NOTCH signaling between ECs and mural cells, CellChat inference suggested enhanced autocrine signaling in ECs that can be activated by disturbed blood flow^48^. Furthermore, signaling to myeloids was increased (Fig. 4i and Extended Data Fig. 6m) in line with the recent implication of NOTCH signaling in macrophage activation and differentiation^49^.

### Fibrosis-associated gene expression profiles

**Fig. 5:**
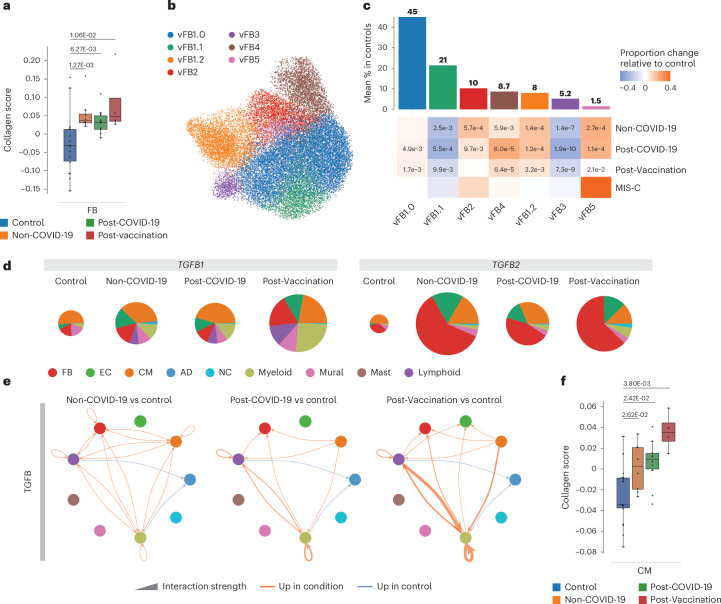
Fibroblast compositional and gene expression changes indicate altered remodeling processes. **a**, Collagen scores across FBs in controls (*n* = 18), Non-COVID-19 (*n* = 8), Post-COVID-19 (*n* = 10) and Post-Vaccination (*n* = 4). Scores were calculated across all expressed collagens. Boxes depict the interquartile range (IQR); horizontal bars indicate the median; whiskers extend to 1.5× IQR; and dots show the value of each patient. *P* values were calculated using the two-sided Wilcoxon rank-sum test and were adjusted for multiple testing (Bonferoni correction). **b**, UMAP embedding delineated seven FB states. **c**, Upper panel, mean abundance (%) of FB states in control left ventricles (*n* = 18). Lower panel, proportional changes of FB states in the four patient groups (Non-COVID-19: *n* = 8, Post-COVID-19: *n* = 10, Post-Vaccination: *n* = 4). Proportional changes are scaled by color: red (increase) and blue (decrease) in disease versus control. *P* values are indicated for significant proportional changes (FDR ≤ 0.05). *P* values were calculated using the two-sided *t*-test based on CLR-transformed values with Benjamini–Hochberg correction. For MIS-C, significance was not calculated due to low sample size (*n* = 2). **d**, Pie charts comparing cell type resolved mean absolute expression levels of *TGFB1* and *TGFB2*. The pie size reflects absolute detection levels; the colors indicate the relative contribution per cell type. Colors indicate cell types; color legend is as in **g**. **e**, Circle plot representations of significantly differential interactions of cell–cell communication for the TGFB pathway (adjusted *P* ≤ 0.05) in patient groups compared to control. *P* values were computed with the one-sided permutation test. The line thickness represents the interaction strength of signals from sending and receiving cells. Color is scaled from zero to maximum in disease versus controls (orange, increased; blue, decreased). The directionality of the signal is indicated with arrows. **f**, Collagen scores across CMs and ECs are as described in **a**. AD, adipocyte; NC, neural cell. Source data

**Extended Data Fig. 7:**
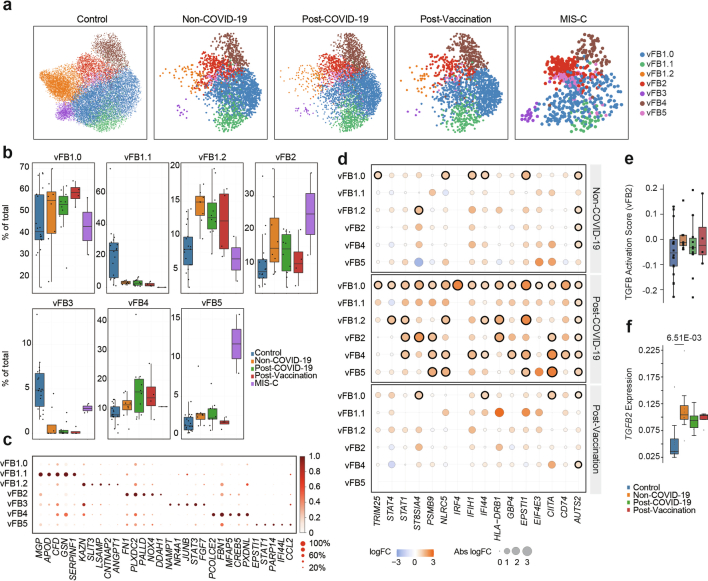
The cellular and molecular signatures in fibroblasts in cardiac inflammation. **a)** Condition-split UMAP showing transcriptional shifts in FB states across patient groups and controls. **b)** Box plots showing FB state frequencies in LV per patient in Non-COVID-19 ( = 8), Post-COVID-19 (n = 10), Post-Vaccination (n = 4), MIS-C (n = 2), and control (n = 18) groups. Boxes depict the interquartile range (IQR), horizontal bars represent the median, whiskers extend to 1.5 × IQR, dots show the value of each patient. *P*-values are shown in Fig. 5c. Dots show proportions per patient. **c)** Dot plot showing the top five marker genes of each FB state. Dot size corresponds to fraction (%) of expressing nuclei; colour indicates scaled mean expression level. **d)** Dot plots showing differential expression of selected interferon-γ response genes in patient groups relative to controls across FB states. Dot colours indicate log2-transformed fold changes (log2FC). Dot sizes indicate absolute log2FC. Black circles indicate significance (FDR ≤ 0.05). Cell types and genes are ordered alphabetically. **e)** TGFB Activation score within the vFB2 state across patient and control groups (Control: n = 18, Non-COVID-19: n = 8, Post-COVID-19: n = 10, Post-Vaccination: n = 4). Boxes depict the interquartile range (IQR), horizontal bars represent the median, whiskers extend to 1.5 × IQR, dots show the value of each patient. P-values were calculated using a two-sided wilcoxon rank-sum test. Dots show scores per patient. **f)** Box plot showing snRNA-seq pseudo-bulk expression levels of *TGFB2* in cardiac tissue from patient and control groups (Control: n = 18, Non-COVID-19: n = 8, Post-COVID-19: n = 10, Post-Vaccination: n = 4). Boxes depict the interquartile range (IQR), horizontal bars represent the median, whiskers extend to 1.5 × IQR, dots show the value of each patient. P-values are indicated for significant changes compared to control, FDR ≤ 0.05. P-values were calculated using the quasi-likelihood F-test and were adjusted for multiple testing (Benjamini Hochberg). Source data

Previous studies reported histopathological findings of fibrosis for post-COVID-19-associated as well as post-vaccination-associated myocarditis^7^. In our patient groups, cardiac MRI showed an increase in late gadolinium enhancement (LGE), which may be a sign of fibrosis development or represents an effect of the increased inflammatory reaction (Extended Data Table 1). snRNA-seq analyses of EMBs did not show a significant increase of total FB numbers (Fig. 1d) but increased collagen gene expression in patient groups compared to control (Fig. 5a). This implied the acquisition of a secretory rather than a proliferative phenotype, as previously observed in dilated cardiomyopathy^13^. Condition-split visualization of the FB latent space supported a transcriptional shift in FB states across patient groups (Extended Data Fig. 7a). Subclustering of FBs identified previously described states (vFB1–4) and one additional state (vFB5) characterized by increased IFN response gene expression (Fig. 5b, Extended Data Fig. 7b,c and Supplementary Tables 12 and 13). vFB1.1 (lipofibroblast) and vFB3 (interacting with MP_OSM^+^) proportions were significantly decreased, whereas vFB4 (extracellular matrix (ECM)-organizing) and vFB5 abundances were increased in all patient groups compared to controls. Interestingly, vFB2 (pro-fibrotic TGFβ-activated) expanded in Non-COVID-19 and Post-COVID-19 but not in Post-Vaccination (Fig. 5c and Extended Data Fig. 7b). GSEA revealed upregulation of IFNγ-stimulated and IFNα-stimulated genes in vFB1.0 and vFB5 in Post-COVID-19 (Extended Data Fig. 7d and Supplementary Table 3). Of note, in MIS-C, vFB2 was not only expanded the most (Extended Data Fig. 7b) but was additionally activated by *TGFβ* (Extended Data Fig. 7e), suggesting a more pronounced fibrotic response compared to the other patient groups.

In cardiac tissue, we detected increased expression of *TGFB1* (Fig. 1f) and *TGFB2* (Extended Data Fig. 7f) encoding TGFB signaling ligands and alterations in their cellular sources of expression across all patient groups compared to controls (Fig. 5d). Myeloid and lymphoid cells contributed greater to the overall *TGFB1* expression levels, particularly myeloid cells in Post-Vaccination and MIS-C and FBs and ECs to the overall *TGFB2* expression levels (Fig. 5d). CellChat analysis suggested upregulated TGFB signaling in all patient groups toward FBs and myeloid and lymphoid cells and additionally to CMs in Non-COVID-19 or to ECs in Post-Vaccination (Fig. 5e). Signaling toward immune cells was especially enhanced in Post-Vaccination. Because of the observed TGFB signaling toward CMs in Non-COVID-19 and ECs in Post-Vaccination, we investigated collagen gene expression in these cell types (Fig. 5h). Although FBs showed the highest expression (Fig. 5a), collagen gene expression was indeed upregulated in CMs (Fig. 5h), suggesting that, besides FBs, there are additional cellular sources for fibrotic gene expression responses.

## Discussion

Our current understanding of the cellular and molecular response mechanisms in cardiac inflammation is mainly based on immunohistochemistry findings in EMBs and studies of autopsy heart tissue samples^2,9,15^. Here we provided single-cell resolved analyses in human EMBs taken during the acute phase of disease from patients with non-COVID-19 lymphocytic myocarditis, myocarditis after SARS-CoV-2 infection and myocarditis after COVID-19 vaccination.

Our results highlighted an important role of *IFNG* expressed in lymphoid cells of post-COVID-19 heart tissue, reflected in ISG expression patterns in multiple cell types and states, and identified upregulated *IL16* and *IL18* expression as hallmarks of post-vaccination myocardial inflammation. This was consistent with previous studies in other organs demonstrating pathogenic IFNγ secretion by recruited T cells and NK cells during SARS-CoV-2 infection^50^ and increased IL16 and 18 serum/plasma levels in anti-COVID-19 mRNA-vaccine-related myocarditis/pericarditis^7,18^. We found these cytokines also expressed during the inflammatory processes within the cardiac microenvironment and, furthermore, noticed differences in the expression of immunosuppressive cytokines, such as TGFB1 and IL34, between groups. These findings suggest a stronger immune response in post-COVID-19 and a more dampened response in post-vaccination myocardial inflammation, further supported by downstream analyses of individual cell types.

Previous studies showed high amounts of CD68^+^ macrophages in cardiac tissue of patients who died or developed myocarditis after SARS-CoV-2 infection or vaccination^2^. In our cohort, both immunohistochemistry and snRNA-seq consistently showed significant expansion of the myeloid cell population. Additionally, snRNA-seq revealed profound compositional changes, including a loss of resident macrophages and an increase in monocytes and monocyte-derived macrophages. These alterations mirrored observations in mice where cardiac injury, myocarditis and stress-induced cardiomyopathy resulted in replacement of resident macrophages by inflammatory monocyte-derived macrophages^51–53^. Notably, the myeloid compositional changes were similar across Non-COVID-19, Post-COVID-19 and Post-Vaccination groups, suggesting that these responses are non-specific.

Among cardiac myeloid states, we did not detect pro-fibrotic transcriptomic signatures as reported for the lungs of patients with COVID-19 or the blood of patients with perimyocarditis after vaccination^7^. However, Post-COVID-19 patients showed pronounced ISG expression in multiple cardiac myeloid states, which suggested augmented inflammatory activity^54^ and was linked to increased stimulation of T cell responses^55^. Additionally, we noted a loss of tissue repair macrophages, such as Lyve1^hi^MHCII^lo^ macrophages, which are critical for repair processes. This shift toward pro-inflammatory macrophages, potentially leading to excessive activation of the adaptive immune system at the expense of repair functions, may contribute to cardiac damage.

Although our observation of macrophage expansion was consistent among snRNA-seq, immunohistochemistry and previous reports, T cell abundances, as determined by the detection of CD3^+^ cells in immunohistochemistry, differed from snRNA-seq. snRNA-seq analyses identified transcriptional profiles corresponding to individual T cells, with abundances in post-COVID-19 and post-vaccination myocarditis similar to those observed in non-COVID-19 lymphocytic myocarditis. Notably, Non-COVID-19 patients were specifically selected based on prior confirmed lymphocytic infiltrations using immunohistochemical detection of CD3^+^ T cells. The sensitivity to detect specific lymphocytic infiltrates by immunohistochemistry is estimated to be only up to 40%^56^. Our data raise the question of whether snRNA-seq may be more sensitive than traditional approaches.

The analysis of snRNA-seq-identified lymphocytes showed increased CD4^+^ to CD8^+^ T cell ratios, fewer activated NK cells and a weaker cytotoxic profile of CD8 T cells post-vaccination compared to post-COVID-19. Although a dominance of cardiac CD4^+^ lymphocytic infiltrates in response to the vaccination was described previously^2,11^, we here show that this aligns with the elevated expression of *IL16*, a cytokine that governs trafficking and biological properties of CD4^+^ T cells^57^. In Post-COVID-19 patients, we identified significantly enriched highly activated CD16^+^CD8^+^ T cells (CD8T_act_effector), not previously reported for cardiac inflammatory responses. Similar T cells in blood from patients with severe COVID-19 were described to be capable of immune-complex-mediated, T cell receptor–independent degranulation and cytotoxicity promoting microvascular EC injury^29^. These findings suggest an over-aggressive cytotoxic lymphocyte response in Post-COVID-19 cardiac tissue in contrast with the more attenuated inflammatory processes in Post-Vaccination patients.

Across patient groups, we found evidence for an inflammatory status of the vasculature, such as heightened expression of *JAM2* and *JAM3*, encoding leukocyte adhesion molecules, and reduced NF-κB inhibitor *NFKBIA* expression, suggesting heightened pro-inflammatory NF-κB signaling. Post-COVID-19 patients additionally exhibited a distinct IFNγ response gene signature in ECs. In hamsters, lung ECs respond to SARS-CoV-2 infection with strong transcriptional pro-inflammatory chemokine responses without evidence for their direct infection^58^. Human cardiac Post-COVID-19 ECs displayed ISG expression patterns similar to those observed in hamster lungs. Vascular permeabilization, endothelial injury and chronic vascular inflammation are central aspects in COVID-19 (ref. ^59^). The known role of IFNγ in regulating endothelial monolayer permeability further supports the presence of endothelial barrier dysfunction in the hearts of these patients.

Increased overall EC abundances, identification of pro-angiogenic tip cells and predicted elevated pro-angiogenic signaling indicated ongoing angiogenesis across patient groups. Similarly, angiogenesis was reported for parvovirus B19–associated myocarditis^60^, potentially aiding in restoring blood supply to affected areas. However, alterations in EC state ratios with a particular shift toward arterial ECs and a decrease in overall mural cell abundances as observed in our cohort may affect vascular network function and maturation. Our findings highlight the need for further investigation of the intricate equilibrium between angiogenesis and inflammation and its implications for cardiac inflammatory pathology.

Overall FB populations were not expanded, but FB states were shifted toward activated vFB2, indicating an acquired secretory phenotype in all patient groups except Post-Vaccination. Interestingly, signs of vFB2 hyperactivation, as identified in end-stage failing human hearts^13^, were observed only in patients with MIS-C, who also exhibited the highest ECM gene expression activity despite their young age. The responses within the FB population were generally less pronounced in Non-COVID-19, Post-COVID19 and Post-Vaccination compared to those in end-stage dilated cardiomyopathy failing hearts^13^.

### Limitations of our study

We would like to emphasise that the EMBs analyzed in this study are extremely difficult to obtain, leading to limited group sizes. However, we employed advanced, state-of-the-art statistical methods suited to effectively manage small sample sizes. Additionally, the patients in this study represent a clinically heterogeneous group, varying in the onset, degree of clinical symptoms and diagnostic evidence. Non-COVID-19 lymphocytic myocarditis cases were selected to clinically match the other disease groups, excluding fulminant myocarditis cases to align with the mild symptoms typical of COVID-19 myocarditis. However, this selection may influence the comparison.

We also cannot determine whether our findings are influenced by differences in the timespan between infection/vaccination and the patient’s hospital admission. On a technical note, we compared EMBs to transmural donor heart controls, as obtaining EMBs from healthy, age-matched controls is impossible, which may affect the sampling of specific cell types. Furthermore, CLR transformation of snRNA-seq data was used to analyze relative compositional changes in cardiac cell types. This method cannot capture absolute values. In contexts like tissue inflammation, where shifts in cell counts are of additional relevance, relative abundance may not fully capture all aspects of the pathology.

Future studies are needed to trace long-term consequences of the here-described changes observed during the acute phase of cardiac inflammation. We expect that our data will advance mechanistic studies to improve treatment and enable preventive strategies and better diagnosis of post-COVID-19 and post-vaccination inflammatory cardiomyopathies.

## Methods

### Patients and ethics statement

This study complies with all ethical regulations associated with human tissue research. Acquisition and use of samples was approved by the Ethics Committee of Berlin and the Ethics Committee of the Charité – Universitätsmedizin Berlin (institutional review board approval numbers EA2/140/16 and EA2/066/20) and conducted in accordance with the Declaration of Helsinki. All subjects gave their written informed consent for their tissues to be collected for research purposes and the data obtained from that research to be published. All patients underwent LV EMB and left heart catheterization after routine non-invasive diagnostic workup and angiography had excluded any specific cause of heart-failure-like symptoms. All EMBs were evaluated histologically and by immunohistochemical stains as well as performing molecular analysis (RT–PCR) for cardiotropic viral genome detection (adenovirus, enteroviruses, human herpes virus 6 (HHV6), parvovirus B19, Epstein–Barr virus and SARS-CoV2) by the Department of Cardiopathology, Institute for Pathology and Neuropathology, University of Tübingen, Germany. Clinical information for the cardiac tissue is available in Extended Data Table 1.

### Cohort samples

Disease samples were collected via LV EMBs from patients with ‘classical’ lymphocytic myocarditis (Non-COVID-19, *n* = 8), patients who clinically recovered from COVID-19 but showed persisting cardiac symptoms indicative of cardiac inflammation (Post-COVID-19, *n* = 10) and patients with cardiac symptoms indicative of cardiac inflammation after vaccination against SARS-CoV-2 (Post-Vaccination, *n* = 4). In addition, we investigated EMBs from patients diagnosed with MIS-C and indicative of cardiac inflammation as well as a follow-up EMB for one of the patients with MIS-C after treatment (MIS-C follow-up). All patients presented with symptoms including chest pain, palpitations, fever, shortness of breath, malaise and/or general weakness and fatigue and an overall increase of cardiac damage-indicating biomarkers (troponin T, NT-pro-BNP, creatine kinase or creatine kinase MB) and CRP levels (Fig. 1a). ECG, echocardiography or signs of recent or ongoing myocardial damage on cardiac MRI ranged from normal or non-specific to borderline low or abnormal and are summarized in Extended Data Table 1. All patients underwent LV EMBs and left heart catheterization after routine non-invasive diagnostic workup and angiography had failed to elucidate any other specific cause of heart failure, such as coronary artery disease.

Patients in the Post-COVID-19 and MIS-C groups were previously tested positive for SARS-CoV-2 infection by nasopharyngeal swap test PCR but tested negative at the timepoint of the actual study. Healthy heart snRNA-seq data were recently generated by us using LV free wall and apical samples from 18 unused donor hearts^12,13^. Cell composition, states and transcript counts across the free wall and apex showed high similarities^13^ and, therefore, are reported grouped together as LV.

### EMB immunhistochemistry

EMBs were fixed in 4% phosphate-buffered formaldehyde and embedded in paraffin. Four-micrometer-thick tissue sections were stained with hematoxylin and eosin and examined by light microscopy. For immunohistological detection of cardiac immune cells, a monoclonal rabbit-anti-CD3 antibody (Novocastra Laboratories, clone SP7, 1:500), a monoclonal mouse anti-human CD68 antibody (DAKO, clone PG-M1, 1:50), a monoclonal rabbit anti-CD4 (Zytomed, clone SP35, 1:50) and a monoclonal mouse anti-CD8 (DAKO, clone C8/144B 1:300) were used. Immunohistochemical analysis was performed on an automated immunostainer following the manufacturer’s protocol (Ventana Medical Systems, Benchmark) and using the ultraView detection system (Ventana Medical Systems) and diaminobenzidine as substrate. Tissue sections were counterstained with hematoxylin. Positive cells were counted using an Axioskop 40 (Zeiss) microscope, and results are given per mm^2^. Representative pictures are shown at ×200 magnification.

### Isolation of cardiac single nuclei from EMBs and processing on the 10x Genomics platform

The isolation of cardiac nuclei and the 10x library preparation were performed at the Max Delbrück Center for Molecular Medicine following our published protocol^61^ with adaptations to low-sized tissue pieces. Next, 1–4-mg-sized flash-frozen cardiac biopsies were placed in a pre-cooled dish and an equally sized droplet of homogenization buffer (250 mM sucrose, 25 mM KCl, 5 mM MgCl_2_, 10 mM Tris-HCl, 1 μM DTT, 1× protease inhibitor, 0.4 U μl^−1^ RNaseIn, 0.2 U μl^−1^ SUPERaseIn and 0.1% Triton X-100 in nuclease-free water) was added. Buffer-encapsulated tissue pieces were sliced with a scalpel. The tissue pieces were then transferred to a 7-ml glass Dounce tissue grinder (Merck), and nuclei were isolated and stained with NucBlue Live ReadyProbes Reagent (Thermo Fisher Scientific). Hoechst^+^ single nuclei were sorted via fluorescence-activated cell sorting (FACS) (BD Biosciences, FACSAria Fusion). The forward scatter (FSC)/side scatter (SSC) gating strategy is shown in Supplementary Fig. 1. Purity and integrity of nuclei were confirmed microscopically, and nuclei numbers were counted using a Countess II (Life Technologies) before processing with the Chromium Controller (10x Genomics) per the manufacturer’s protocol. Single-nucleus 3′ gene expression libraries were created using version 3.1 Chromium Single Cell Reagent Kits (10x Genomics) following the manufacturerʼs instructions. cDNA library quality control was performed using Bioanalyzer High Sensitivity DNA Analysis (Agilent Technologies) and a KAPA Library Quantification Kit. cDNA libraries were sequenced on an Illumina NovaSeq with a targeted read number of 30,000–50,000 reads per nucleus (Supplementary Fig. 1b–d).

### Sequencing data pre-processing and transcriptome mapping

The binary base call (BCL) sequence files were converted to FASTQ format using bcl2fastq (version 2.20). Mapping of sequencing reads from each sample to a modified pre-mRNA version of the human reference genome (version GRCh38, .gtf file from Ensembl release 84) was performed using the Cell Ranger suite (version 3.0.2). The Cell Ranger reference file was created following the instructions from the 10x Genomics website (https://www.10xgenomics.com/support/single-cell-gene-expression) and the specifications provided in the DCMheart GitHub repository (https://github.com/heiniglab/DCM_heart_cell_atlas/)^13^. Reads mapping to exonic and intronic regions were counted. Sequencing mapping quality was assessed using Cell Ranger summary statistics. Reads mapping multiple genome features were discarded.

### Count data and data matrix annotation

The identification of empty droplets was performed using emptyDrops, implemented in the Cell Ranger workflow. The filtered_feature_bc_matrix.h5 files and the metadata information were integrated into an annotated data object (AnnData).

### Annotated data quality control, batch correction and clustering

Quality control and downstream analyses of the concatenated annotated matrices were performed using the Single-Cell Analysis in Python toolkit Scanpy (version 1.5.1)^62,63^. Doublet identification and removal was performed using Solo (version 0.3)^64^. Scrublet scores with prior log transformation were used as an independent doublet detection and filtering method (version 0.2.1)^65^. Nuclei with n_counts (≤300), genes (300 ≤ n_genes ≤ 5,000), mitochondrial genes (≤1%), ribosomal genes (percent_ribo ≤ 1%) and softmax Solo scores (≤0.5) were excluded from downstream analyses.

Normalized and log-transformed read counts were used to identify highly variable genes. The data were corrected and scaled to unit variance to account for the effects of the percentage of mitochondrial genes expressed. To select the principal axes of variation, denoise the data and compute the neighborhood relations of cells, a dimensionality reduction using principal component analysis (PCA) and the elbow methods were used. To remove potential batch effects within our data, before the dimensionality reduction using the UMAP method, principal components were adjusted using Harmony^66^ with ‘Patient’ as batch_key. Clustering of nuclei using the community detection based on optimizing the modularity algorithm Leiden^67,68^ was performed. Next, differential gene expression analyses were performed using the two-sided Wilcoxon rank-sum test, and clusters displaying low differences in their gene expression profiles were merged. Nuclei were assigned to cell types and posteriorly subclustered to identify cell states. The cell state annotation step revealed nuclei, denoted as unassigned in Fig. 1c (*n* = 11,832 ‘Nuclei’; 5.8%), that correspond to droplets with chimeric transcriptional profiles. Whether these droplets represent true data, background noise or multiplets is unclear.

### Differential gene expression analysis

DEGs per cell type and state were calculated using the two-sided Wilcoxon rank-sum test as implemented in Scanpy^62^. Testing for DEGs was done using log-transformed and normalized to library size count values. Only genes with mean expression greater than 0.0125 were considered. Genes with a false discovery rate (FDR) < 0.05 and absolute log_2_ fold change (FC) > 0.5 were considered differentially expressed. For rare cell states (≤3 samples with ≤5 nuclei), DEG differences were not calculated.

To identify disease-specific expression profiles, DEG analyses were performed between control and myocarditis groups. For this, transcript counts per gene of all nuclei for a given sample (tissue level), cluster (cell type level) or subcluster (state level) from the same individual were aggregated to create ‘pseudo-bulk’ samples. Testing for DEGs in pseudo-bulks was performed using the empirical Bayes quasi-likelihood *F*-test function (glmQLFtest) available in the R package edgeR (version 3.28.1)^69,70^.

### Compositional analysis

To identify disease-specific changes in the proportion of cells between control and disease groups, a CLR-based approach excluding unassigned nuclei was used^13^ to account for the compositional nature of the data. For this, count data were transformed using CLR transformation. Zeros in the data, assumed to result from insufficient sampling depth, were imputed using the multiplicative replacement method^71^. Statistical differences between disease groups and controls were assessed by fitting a linear model to the CLR-transformed values, with the group encoded as an indicator variable. Significance was determined using a two-sided *t*-test on the regression coefficient. Differential abundance of all cell types or states was analyzed separately for each disease group compared to controls. For cell state analyses within each cell type, only the counts assigned to that specific cell type were considered, effectively normalizing cell states within each type to 100%. Samples with fewer than 10 cells per cell type were excluded from the cell state analysis. For analysis of lymphoid state abundances, samples with fewer than 30 total lymphoid cells were excluded.

In addition to CLR values, abundance differences were expressed as the mean percentage differences between groups, and statistical significance was determined from the CLR-transformed values. Positive values indicate higher abundance in the disease group. *P* values were adjusted for multiple testing using the Benjamini–Hochberg method, and only significant results are reported.

EC_PKHD1L1^+^ comprised mostly endocardial (EC7) and few lymphatic (EC8) ECs (Supplementary Fig. 2b). We noticed that controls barely, and EMB samples to a variable extent, contributed to this cluster, likely due to a sampling bias when collecting EMBs compared to transmural sections (Supplementary Fig. 2b). We, therefore, decided to exclude EC_PKHD1L1^+^ from analyses where amounts may influence results.

At this point, samples with fewer than 10 nuclei per cell type were excluded from the analyses. To calculate compositional differences of lymphocyte states, only samples with a minimum of 30 nuclei were included to account for the high cellular heterogeneity within the lymphoid cell population. Differences in mean percentages between groups were reported using the statistical significance obtained in the CLR step. For interpretation, disease groups with positive values suggest an increase in abundance. Benjamini–Hochberg correction for multiple testing was applied (only significant results are shown).

### GO enrichment analysis and GSEA

GO and GSEA analyses^72,73^ were performed using GSEApy version 0.10.5—a Python implementation for Enrichr with default settings^74^. GO analyses were performed with the gene set libraries ‘GO_Biological_Process_2021’ and ‘KEGG_2021_Human’, and DEGs identified per cell type or state were used as input. GSEAs were performed with the collections of gene sets in the Molecular Signatures Database ‘MSigDB_Hallmark_2020’, and DEGs identified per condition on the tissue, cell type or cell state level, respectively, were used as input. Gene background was defined using all genes that were expressed in the given cell type or cell state (mean expression > 0.0125).

### Gene set score enrichment

The score_genes functionality implemented in Scanpy was used on log-transformed and scaled transcript counts to compute enrichment of individual gene sets^62^. The MHC-I score was based on the expression of MHC-I genes in aggregated non-immune cardiac cells (excluding lymphoids, myeloids and mast cells as well as unassigned, EC_PKHD1L1^+^, adipocytes and neural cells). The collagen score was based on all expressed collagens per cell type. TGFB activation score was based on a list of genes curated from ref. ^75^ with logFC > 0.7 and FDR < 0.05. The cytotoxic^76^ and cytotoxic cytokine^77^ scores were calculated using Seurat’s (version 5.1) AddModuleScore function^78^ on the log-transformed and normalized counts from aggregated CD4^+^ or CD8^+^ T cells. Patients with fewer than five CD4^+^ or CD8^+^ T cells were excluded from the analysis.

### Intercellular signaling cross-talk and differential cell–cell interaction network analysis

To infer, visualize and analyze intercellular communication among all assigned cell types and cell states, we used CellChat, an open-source R package (version 1.6.1, http://www.cellchat.org/cellchatdb/)^79^. We first excluded genes expressed in less than 1% of the nuclei per cell state before running CellChat. We then used the CellChat database (http://www.cellchat.org/cellchatdb/) and log-transformed normalized gene counts to identify and select overexpressed signaling genes (default parameters). Due to the differences in abundances across cell states, we computed the communication probabilities considering the cell state’s population size. We excluded cell–cell communication if a cell state had fewer than 10 nuclei. The communication probabilities on signaling pathways were filtered using *P* ≤ 0.05. We used network centrality measures in all inferred pathway communication networks (default parameters) to identify cell states that act as dominant senders, receivers, mediators and influencers. We compared the differences in the results obtained for cell–cell interactions from control and medical conditions to highlight significant changes.

Pathway-specific differences in interaction strength between control and diseases across cell types were calculated using the communication probabilities per cell state and CellChat’s aggregateNet() function. We then aggregated the communication probabilities per cell state from the same cell type using the mergeInteractions() and plotted these data using netVisual_diffInteraction() CellChat functions.

### Statistics and reproducibility

Replicates and statistical tests are described in the figure legends. Sample sizes were not predetermined using statistical methods. No sample size calculations were performed. Sample size was governed by tissue availability, and input tissue mass was on the basis of endomyocardial biopsy size. No samples were excluded. All snRNA-seq analyses including clustering were done using unbiased techniques. Investigators were blinded to the groups when performing initial analyses.

### Reporting summary

Further information on research design is available in the Nature Portfolio Reporting Summary linked to this article.

## Supplementary information

Supplementary Fig. 1

Reporting Summary

**Supplementary Table 1** Cell type marker list. The top 100 DEGs were determined using a two-sided Wilcoxon rank-sum test. *P* values were adjusted for multiple testing using Benjamini–Hochberg correction. FCs are provided as log_2_FCs. **Supplementary Table 2** DEGs between studied conditions using a pseudo-bulk approach across all nuclei per patient. *P* values were calculated using edgeRʼs quasi-likelihood *F*-test. *P* values were adjusted for multiple testing using Benjamini–Hochberg correction. FCs and significance values are provided for all annotated genes in the human genome. **Supplementary Table 3** Significantly dysregulated pathways per cell type and states as determined by GSEApy. The MSigDB Hallmark (2020) database was used as a pathway database. Only significant pathways are provided, determined using GSEAʼs Fisherʼs exact test. *P* values were adjusted for multiple testing using Benjamini–Hochberg correction. **Supplementary Table 4** Myeloid cell state marker list. The top 100 DEGs were determined using a two-sided Wilcoxon rank-sum test. *P* values were adjusted for multiple testing using Benjamini–Hochberg correction. FCs are provided as log_2_FCs. **Supplementary Table 5** DEGs between studied conditions using a pseudo-bulk approach across all myeloid nuclei (cell type) and per myeloid cell state. *P* values were calculated using edgeRʼs quasi-likelihood *F*-test. Only significantly dysregulated genes after multiple testing correction (Benjamini–Hochberg) are provided. **Supplementary Table 6** Lymphoid cell state marker list. The top 100 DEGs were determined using a two-sided Wilcoxon rank-sum test. *P* values were adjusted for multiple testing using Benjamini–Hochberg correction. FCs are provided as log_2_FCs. **Supplementary Table 7** DEGs between studied conditions using a pseudo-bulk approach across all lymphoid nuclei (cell type) and per lymphoid cell state. *P* values were calculated using edgeRʼs quasi-likelihood *F*-test. Only significantly dysregulated genes after multiple testing correction (Benjamini–Hochberg) are provided. **Supplementary Table 8** CM cell state marker list. The top 100 DEGs were determined using a two-sided Wilcoxon rank-sum test. *P* values were adjusted for multiple testing using Benjamini–Hochberg correction. FCs are provided as log_2_FCs. **Supplementary Table 9** DEGs between studied conditions using a pseudo-bulk approach across all CMs (cell type) and per CM cell state. *P* values were calculated using edgeRʼs quasi-likelihood *F*-test. Only significantly dysregulated genes after multiple testing correction (Benjamini–Hochberg) are provided. **Supplementary Table 10** Cell state marker list of vascular cells (ECs, PCs and SMCs). The top 100 DEGs were determined using a two-sided Wilcoxon rank-sum test. *P* values were adjusted for multiple testing using Benjamini–Hochberg correction. FCs are provided as log_2_FCs. **Supplementary Table 11** DEGs between studied conditions using a pseudo-bulk approach across all vascular cell type (cell type) and per vascular cell state. *P* values were calculated using edgeRʼs quasi-likelihood *F*-test. Only significantly dysregulated genes after multiple testing correction (Benjamini–Hochberg) are provided. **Supplementary Table 12** Fibroblast cell state marker list. The top 100 DEGs were determined using a two-sided Wilcoxon rank-sum test. *P* values were adjusted for multiple testing using Benjamini–Hochberg correction. FCs are provided as log_2_FCs. **Supplementary Table 13** Cell state marker list of FBs. The top 100 DEG were determined using a two-sided Wilcoxon rank-sum test. *P* values were adjusted for multiple testing using Benjamini–Hochberg correction. FCs are provided as log_2_FCs.

## Source data

Source Data Fig. 1 Statistical Source Data.

Source Data Fig. 2 Statistical Source Data.

Source Data Fig. 3 Statistical Source Data.

Source Data Fig. 4 Statistical Source Data.

Source Data Fig. 5 Statistical Source Data.

Source Data Extended Data Fig. 1 Uncropped figures.

Source Data Extended Data Fig. 1 Statistical Source Data.

Source Data Extended Data Fig. 2 Statistical Source Data.

Source Data Extended Data Fig. 3 Statistical Source Data.

Source Data Extended Data Fig. 4 Statistical Source Data.

Source Data Extended Data Fig. 5 Statistical Source Data.

Source Data Extended Data Fig. 6 Statistical Source Data.

Source Data Extended Data Fig. 7 Statistical Source Data.

## Figures and Tables

**Extended Data Table 1:**
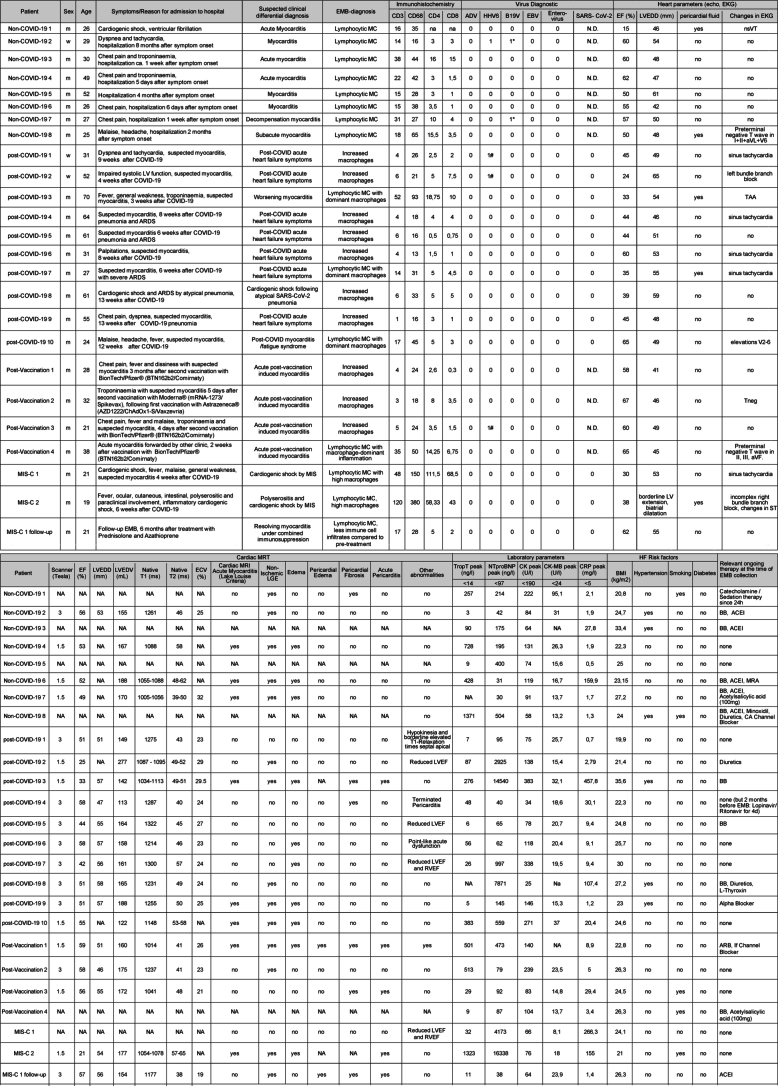
Clinical metadata of patients

Patient information, clinical metadata and laboratory values.

**Extended Data Table 2:**
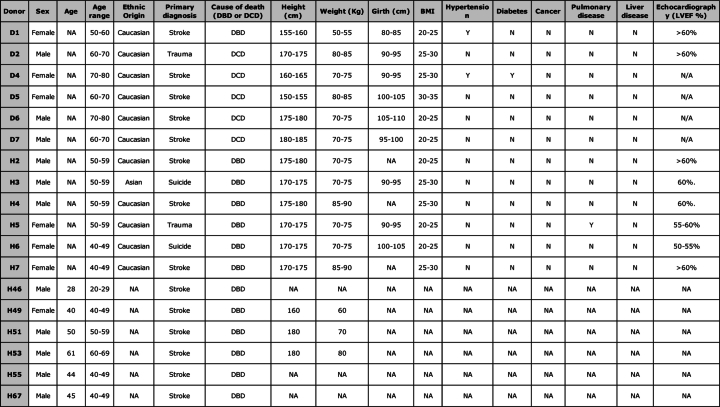
Clinical metadata of controls

Control group information and clinical metadata.

## Data Availability

All data generated and analyzed in this study have been deposited at the European Genome-phenome Archive (EGA) (https://ega-archive.org), which is hosted by the European Bioinformatics Institute and the Centre for Genomic Regulation, under accession number EGAS50000000769. Raw data can be downloaded from the EGA after completing the data access agreement (DAA) of the Max Delbrück Center. The DAA is in place to ensure that all users who request data adhere to the General Data Protection Regulation of the European Union and to protect the confidentiality of the research participants. Instructions on how to register with the EGA and how to access the data are available at https://ega-archive.org/access/request-data/how-to-request-data/. Processed single-nucleus transcriptomic data will be available through the CELLxGENE platform in the h5ad format (https://cellxgene.cziscience.com/collections/328d71f0-0ed7-4518-966f-be6bd0797324) and on Zenodo (https://zenodo.org/records/14258362). Metadata sheets and patient information are available in Extended Data Table 1.

## Code availability

All code used to generate the figures in this publication are available on GitHub (https://github.com/Norbert-Hubner-Lab/Heart_Biopsy_Covid). All scripts run on Jupyter notebooks are available as .ipynb files, and scripts executed in the command line are available as .txts or .sh files. R scripts are available as .R. Anaconda environments are available as .yml files containing information on package versions.
