# Supplementary material for: The cellular and molecular cardiac tissue responses in human inflammatory cardiomyopathies after SARS-CoV-2 infection and COVID-19 vaccination
Source: Nat Cardiovasc Res. 2025 Feb 24;4(3):330–45. doi: 10.1038/s44161-025-00612-6 (PMC11913730; doi:10.1038/s44161-025-00612-6)
Supplement: Supplementary file 1 — Supplementary Fig. 1 [file 44161_2025_612_MOESM1_ESM.pdf]

nature cardiovascular research

Supplementary information https://doi.org/10.1038/s44161-025-00612-6

The cellular and molecular cardiac tissue responses in human inflammatory cardiomyopathies after SARS-CoV-2 infection and COVID-19 vaccination

In the format provided by the authors and unedited

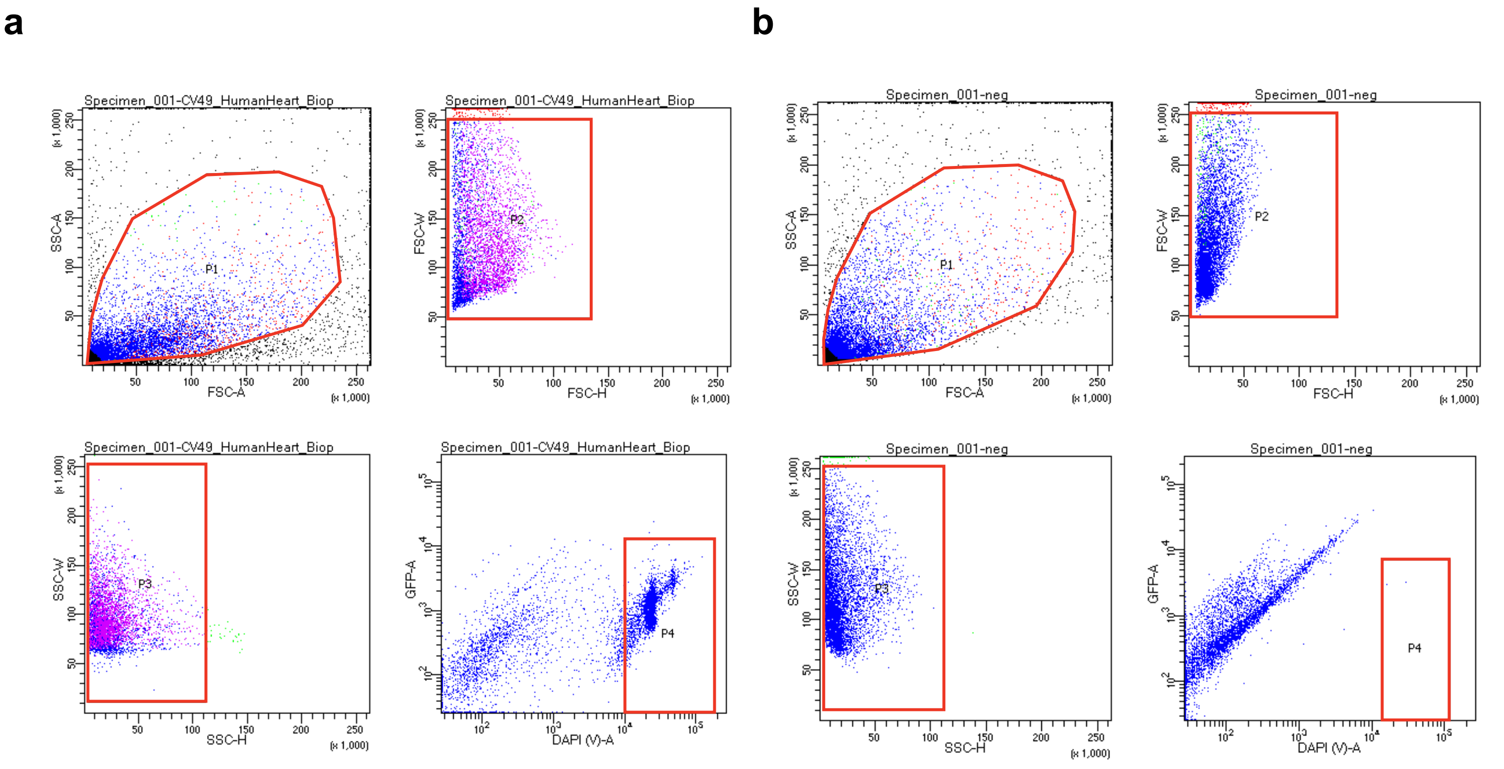

Tube: CV49_HumanHeart_Biop

Population	#Events	%Parent	%Total
All Events	10,000	####	100.0
P1	5,590	55.9	55.9
P2	4,771	85.3	47.7
P3	4,682	98.1	46.8
P4	2,659	56.8	26.6

Tube: neg

Population	#Events	%Parent	%Total
All Events	10,000	####	100.0
P1	5,827	58.3	58.3
P2	5,526	94.8	55.3
P3	5,351	96.8	53.5
P4	2	0.0	0.0

Supplementary Figure 1: FACS gating strategy for removal of cell debris and purification of nuclei. a) Size gating to remove doublets and aggregates was applied (FSC-A, SSC-A, FSC-H, FSC-W, SSC-W, SSC-H), followed by sorts for Hoechst-positive nuclei (purple population P4). b) Example of negative control (no NucBlue Live ReadyProbes Reagent applied) with the same parameters as in a).
