# Supplementary material for: The cellular and molecular cardiac tissue responses in human inflammatory cardiomyopathies after SARS-CoV-2 infection and COVID-19 vaccination
Source: Nat Cardiovasc Res. 2025 Feb 24;4(3):330–45. doi: 10.1038/s44161-025-00612-6 (PMC11913730; doi:10.1038/s44161-025-00612-6)
Supplement: Supplementary file 2 — Reporting Summary [file 44161_2025_612_MOESM2_ESM.pdf]

nature portfolio

Corresponding author(s): Norbert Hubner

Last updated by author(s): 01/07/2025

Reporting Summary

Nature Portfolio wishes to improve the reproducibility of the work that we publish. This form provides structure for consistency and transparency in reporting. For further information on Nature Portfolio policies, see our Editorial Policies and the Editorial Policy Checklist.

Statistics

For all statistical analyses, confirm that the following items are present in the figure legend, table legend, main text, or Methods section.

n/a	Confirmed	
☐	☒	The exact sample size (*n*) for each experimental group/condition, given as a discrete number and unit of measurement
☐	☒	A statement on whether measurements were taken from distinct samples or whether the same sample was measured repeatedly
☐	☒	The statistical test(s) used AND whether they are one- or two-sided<br>*Only common tests should be described solely by name; describe more complex techniques in the Methods section.*
☐	☒	A description of all covariates tested
☐	☒	A description of any assumptions or corrections, such as tests of normality and adjustment for multiple comparisons
☐	☒	A full description of the statistical parameters including central tendency (e.g. means) or other basic estimates (e.g. regression coefficient) AND variation (e.g. standard deviation) or associated estimates of uncertainty (e.g. confidence intervals)
☐	☒	For null hypothesis testing, the test statistic (e.g. *F*, *t*, *r*) with confidence intervals, effect sizes, degrees of freedom and *P* value noted<br>*Give P values as exact values whenever suitable.*
☒	☐	For Bayesian analysis, information on the choice of priors and Markov chain Monte Carlo settings
☐	☒	For hierarchical and complex designs, identification of the appropriate level for tests and full reporting of outcomes
☒	☐	Estimates of effect sizes (e.g. Cohen's *d*, Pearson's *r*), indicating how they were calculated

Our web collection on statistics for biologists contains articles on many of the points above.

Software and code

Policy information about availability of computer code

Data collection	Cellranger (v3.0.2); bcl2fastq (v2.20); Single-Cell Analysis in Python toolkit Scanpy (version 1.5.1); Solo (v0.3); Scrublet (v0.2.1)
Data analysis	All code used to generate the figures in this publication are available on GitHub (https://github.com/Norbert-Hubner-Lab/Heart_Biopsy_Covid). All scripts run on jupyter notebooks are available as ".ipynb" files, and scripts executed in command line are available as .txts or .sh files. R scripts are available as ".R". Anaconda environments are available as yml files containing information on package versions.

For manuscripts utilizing custom algorithms or software that are central to the research but not yet described in published literature, software must be made available to editors and reviewers. We strongly encourage code deposition in a community repository (e.g. GitHub). See the Nature Portfolio guidelines for submitting code & software for further information.

Data

Policy information about availability of data

All manuscripts must include a data availability statement. This statement should provide the following information, where applicable:

- Accession codes, unique identifiers, or web links for publicly available datasets
- A description of any restrictions on data availability
- For clinical datasets or third party data, please ensure that the statement adheres to our policy

All data generated and analyzed in this study have been deposited at the European Genome-phenome Archive (https://ega-archive.org), which is hosted by the EBI

and the CRG, under accession number (EGAS50000000769). Processed single-nucleus transcriptomic data will be available through the cellxgene platform in the h5ad format (https://cellxgene.cziscience.com/collections/328d71f0-0ed7-4518-966f-be6bd0797324) and on Zenodo (https://zenodo.org/records/14258362). Metadata sheets and patient information are available in Extended Data Table 1.

Research involving human participants, their data, or biological material

Policy information about studies with human participants or human data. See also policy information about sex, gender (identity/presentation), and sexual orientation and race, ethnicity and racism.

Reporting on sex and gender	Sex and gender based analyses have not been performed due to limited numbers of individuals investigated.
Reporting on race, ethnicity, or other socially relevant groupings	Not reported due to limited number of individuals investigated
Population characteristics	Our clinical cohort consisted of i) "classical" lymphocytic myocarditis patients (Non-COVID-19, n=8), ii) patients with signs of acute myocarditis following SARS-CoV-2 infection (Post-COVID-19, n=10), iii) patients with signs of acute myocarditis following vaccination against COVID-19 (Post-Vaccination, n=4), iv) MIS-C patients with signs of acute myocarditis (n=2), and v) control donor left ventricular tissue that we have analysed previously. All patients presented with symptoms including chest pain, palpitations, fever, shortness of breath, malaise, and/or general weakness and fatigue, and an overall increase of cardiac damage indicating biomarkers (troponin T, NT-pro-BNP, creatine kinase, or creatine kinase-MB) and CRP levels. ECG, echocardiography, or signs of recent or ongoing myocardial damage in cardiac magnetic resonance imaging ranging from normal or nonspecific to borderline low or abnormal are summarised in Extended Data Table 1. All patients underwent left ventricular EMBs and left heart catheterization after routine non-invasive diagnostic work-up and angiography had failed to elucidate any other specific cause of heart failure such as coronary artery disease. Post-COVID-19 and MIS-C patients were previously tested positive for SARS-CoV2 infection by nasopharyngeal swab test PCR. Most Post-Vaccination patients experienced symptom onset within days after the second dose of the vaccine. Consistent with prior reports, the cohort was predominantly male (87.5%; Non-COVID-19: 87.5%, Post-COVID-19: 80%, Post-Vaccination: 100%, and MIS-C: 100%) with an average age of 37 ± 16 years (ranging from 19 to 70 years). The age of the two MIS-C patients was 20 and 21 years. Post-COVID-19 patients were slightly older than the other patients (Fig. 1a; Extended Data Table 1). Selection of Non-COVID-19 patients was based on positive EMB results showing lymphocytic myocarditis and similarities in sex and age compared to the other disease groups. In the MIS-C group, one patient underwent an additional EMB, 6 months following combined immunosuppression with prednisolone and azathioprine.<br>Clinical histopathology and immunostaining on EMB identified significant widespread increased interstitial macrophage infiltration in all patients and additionally lymphocytic myocarditis in 30% of Post-COVID-19, 25% of Post-Vaccination and 100% of MIS-C patients (Extended Data Table 1, Extended Data Fig. 1a,b). Our observations are in agreement with previous reports, where the majority of Post-COVID-19 and mRNA vaccinated patients with signs of myocarditis showed predominantly macrophage infiltrates into the myocardium. No SARS-CoV-2 genome was detected by PCR in EMBs of Post-COVID-19 and MIS-C patients. EMBs that were not used for diagnostic workup were included for snRNA-seq analyses to investigate the cellular and molecular changes of myocardial inflammatory responses across the different disease entities.
Recruitment	We have addressed this in detail in the limitations section.<br>The endomyocardial biopsies (EMBs) analyzed in this study are extremely difficult to obtain, leading to limited group sizes. Additionally, the patients in this study represent a clinically heterogeneous group, varying in the onset, degree of clinical symptoms, and diagnostic evidence. Non-COVID-19 lymphocytic myocarditis cases were selected to clinically match the other disease groups, excluding fulminant myocarditis cases to align with the mild symptoms typical of COVID-19 myocarditis.
Ethics oversight	This study complies with all ethical regulations associated with human tissue research. Acquisition of samples was approved by the Ethics Committee of Berlin and the Ethics Committee of the Charité – Universitätsmedizin Berlin (IRB approval number: EA2/140/16 and EA2/066/20) and conducted in accordance with the Declaration of Helsinki. All subjects gave their informed consent for inclusion before they participated in the study.

Note that full information on the approval of the study protocol must also be provided in the manuscript.

Field-specific reporting

Please select the one below that is the best fit for your research. If you are not sure, read the appropriate sections before making your selection.

☒ Life sciences ☐ Behavioural & social sciences ☐ Ecological, evolutionary & environmental sciences

For a reference copy of the document with all sections, see nature.com/documents/nr-reporting-summary-flat.pdf

Life sciences study design

All studies must disclose on these points even when the disclosure is negative.

Sample size	Limited availability of cardiac biopsies; Group size estimations based on previous studies (Reichart, Lindberg, Maatz et al, Science 2022).
Data exclusions	No data has been excluded from analyses
Replication	Several patients for each group were included in this study, yielding robust statistically significant findings. No further patients and specimens

Replication	are available.
Randomization	Cardiac biopsies were obtained in the hospital from patients with clinical signs of myocarditis. Biopsies were sent to pathology and stored in a biobank. All myocarditis samples in this study were randomized and blinded before biopsies were further processed and snRNAseq analyses were carried out. Data from healthy donor controls were taken from previously published data (Reichart et al, Science 2022). Harmonization of data was carried out at the patient level. After the first AnnData objects were generated, the study was unblinded to carry out group comparisons.
Blinding	At the time cardiac biopsies were taken, blinding was not possible since a strict clinical indication for such an invasive procedure is necessary based on the symptoms of the patient. Subsequently and before further sample processing of all myocarditis samples investigators were blinded. After the first AnnData object was generated, the study was unblinded to carry out group comparisons.

Reporting for specific materials, systems and methods

We require information from authors about some types of materials, experimental systems and methods used in many studies. Here, indicate whether each material, system or method listed is relevant to your study. If you are not sure if a list item applies to your research, read the appropriate section before selecting a response.

Materials & experimental systems

n/a	Involved in the study
☐	☒ Antibodies
☒	☐ Eukaryotic cell lines
☒	☐ Palaeontology and archaeology
☒	☐ Animals and other organisms
☒	☐ Clinical data
☒	☐ Dual use research of concern
☒	☐ Plants

Methods

n/a	Involved in the study
☒	☐ ChIP-seq
☐	☒ Flow cytometry
☒	☐ MRI-based neuroimaging

Antibodies

Antibodies used	For immunohistological detection of cardiac immune cells, a monoclonal rabbit-anti-CD3 antibody (Clone SP7, 1:500, Novocastra Laboratories, Newcastle upon Tyne, GB), a monoclonal mouse anti-human CD68 antibody (Clone PG-M1, 1:50, DAKO), a monoclonal rabbit anti-CD4 (clone SP35, 1:50, Zytomed) and a monoclonal mouse anti-CD8 (clone C8/144B 1:300, DAKO) were used.
Validation	Monoclonal mouse anti-human CD68 antibody (Clone PG-M1, DAKO): "Labels human monocytes and macrophages. The antibody is of value for demonstration of monocytes and macrophages in normal and pathological specimens".<br>Monoclonal rabbit anti-CD4 (clone SP35, Zytomed):"The antibody is used for the specific localization of CD4 in tissue sections of formalin-fixed, paraffin-embedded tissue and in frozen sections. For use as an in vitro diagnostic tool."https://www.zytomed-systems.de/assets/datasheets/GA_BRB042_DE_V01_Gef.pdf<br>Monoclonal mouse anti-CD8 (clone C8/144B, DAKO): "Monoclonal Mouse Anti-Human, Ready-to-use antibody, Unconjugated, Immunohistochemistry. Synthetic peptide corresponding to the 13 C-terminal amino acids of cytoplasmic domain of human CD8α coupled to thyroglobulin. CD8 is a 68 kDa transmembrane glycoprotein expressed as a heterodimer by a majority of thymocytes, and by class I major histocompatibility complex restricted, mature, suppressor/cytotoxic T cells".

Plants

Seed stocks	*Report on the source of all seed stocks or other plant material used. If applicable, state the seed stock centre and catalogue number. If plant specimens were collected from the field, describe the collection location, date and sampling procedures.*
Novel plant genotypes	*Describe the methods by which all novel plant genotypes were produced. This includes those generated by transgenic approaches, gene editing, chemical/radiation-based mutagenesis and hybridization. For transgenic lines, describe the transformation method, the number of independent lines analyzed and the generation upon which experiments were performed. For gene-edited lines, describe the editor used, the endogenous sequence targeted for editing, the targeting guide RNA sequence (if applicable) and how the editor was applied.*
Authentication	*Describe any authentication procedures for each seed stock used or novel genotype generated. Describe any experiments used to assess the effect of a mutation and, where applicable, how potential secondary effects (e.g. second site T-DNA insertions, mosiacism, off-target gene editing) were examined.*

Flow Cytometry

Plots

Confirm that:

☒ The axis labels state the marker and fluorochrome used (e.g. CD4-FITC).

☒ The axis scales are clearly visible. Include numbers along axes only for bottom left plot of group (a 'group' is an analysis of identical markers).

☒ All plots are contour plots with outliers or pseudocolor plots.

☒ A numerical value for number of cells or percentage (with statistics) is provided.

Methodology

Sample preparation	Hoechst-positive single nuclei were sorted via FACS (BD Biosciences: Influx, XDP, or FACSAria, for gating strategy see Supplementary Figure 1). Purity and integrity of nuclei were confirmed microscopically and nuclei numbers were counted using a Countess II (Life | Technologies) before processing with the Chromium Controller (10X Genomics) per the manufacturer's protocol.
Instrument	BD FACSAria Fusion
Software	BD FACSDiva 9.01
Cell population abundance	26% of total nuclei per samples was recovered. Purity and integrity of nuclei were confirmed microscopically and nuclei numbers were counted using a Countess II (Life | Technologies) before processing with the Chromium Controller (10X Genomics) per the manufacturer's protocol.
Gating strategy	Size gating to remove doublets and aggregates was applied (FSC-A, SSC-A, FSC-H, FSC-W, SSC-W, SSC-H), followed by sorts for Hoechst-positive nuclei (population P4).

☒ Tick this box to confirm that a figure exemplifying the gating strategy is provided in the Supplementary Information.
