# Supplementary material for: The cellular and molecular cardiac tissue responses in human inflammatory cardiomyopathies after SARS-CoV-2 infection and COVID-19 vaccination
Source: Nat Cardiovasc Res. 2025 Feb 24;4(3):330–45. doi: 10.1038/s44161-025-00612-6 (PMC11913730; doi:10.1038/s44161-025-00612-6)

Separated uncropped files for images in ED_Figure_1

ED_Figure_1a - Non-COVID-19 HE

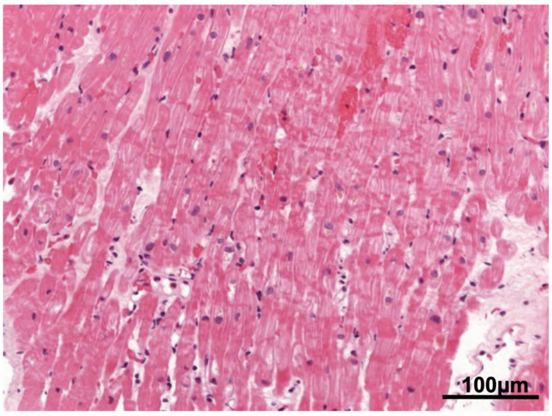

ED_Figure_1a - Non-COVID-19 CD3

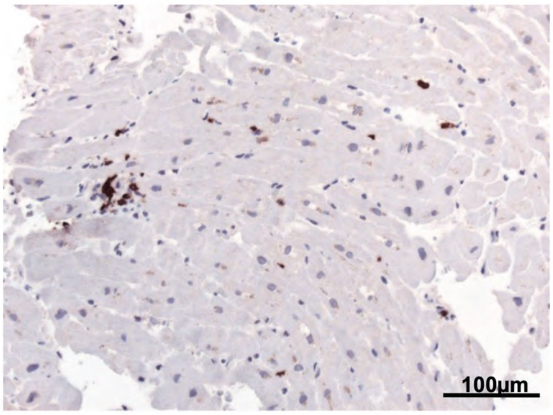

ED_Figure_1a - Non-COVID-19 CD68

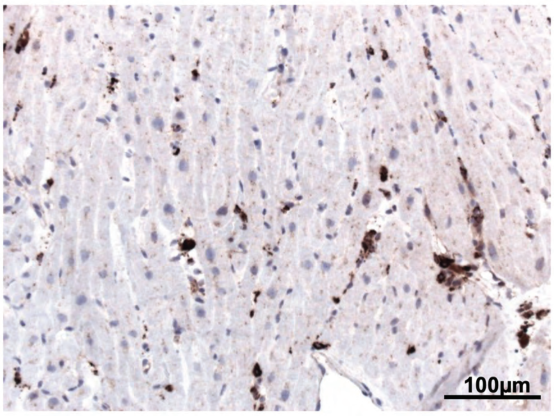

ED_Figure_1a - Post-COVID-19 HE

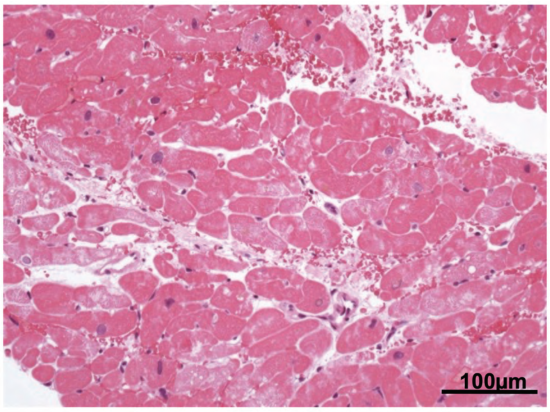

ED_Figure_1a - Post-COVID-19 CD3

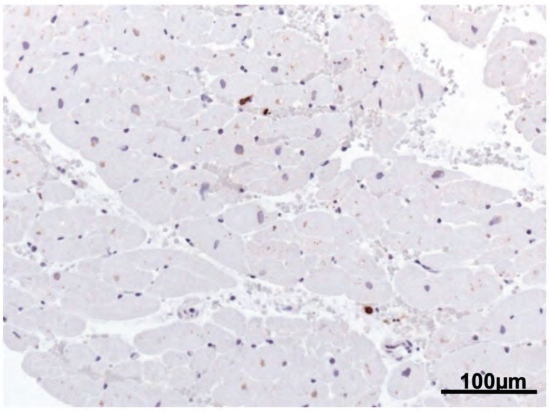

ED_Figure_1a - Post-COVID-19 CD68

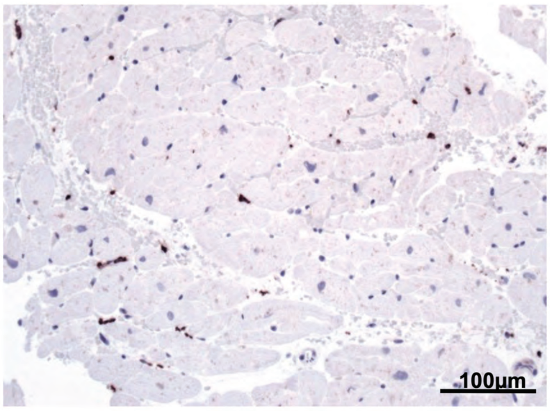

ED_Figure_1a - Post-Vaccination HE

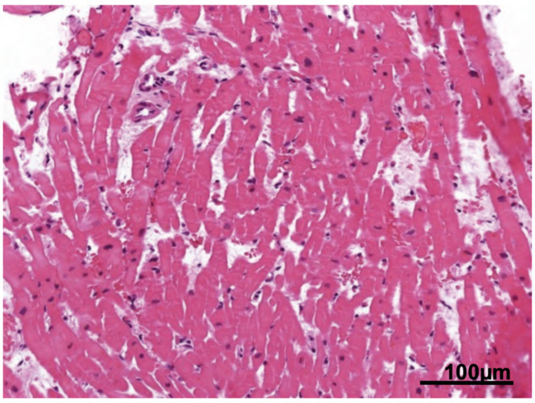

ED_Figure_1a - Post-Vaccination CD3

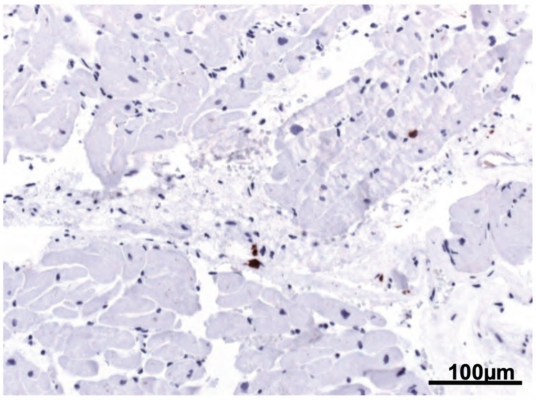

ED_Figure_1a - Post-Vaccination CD68

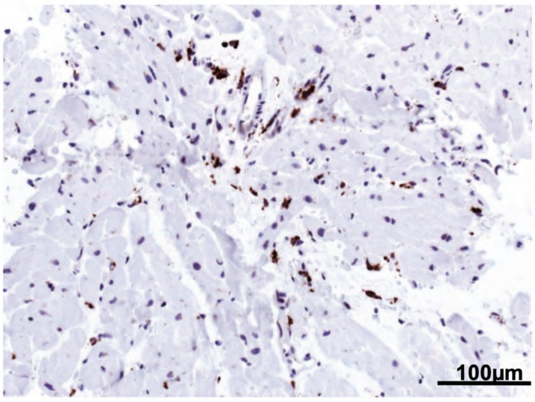

ED_Figure_1a - MIS-C HE

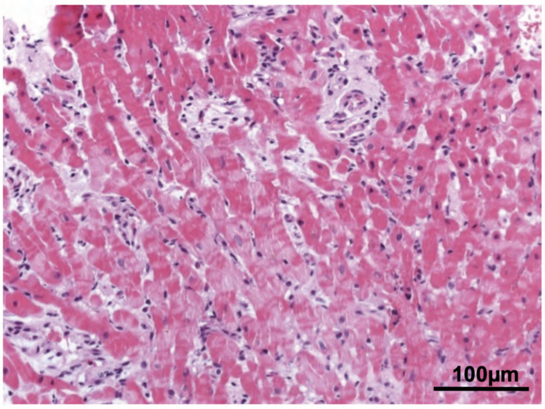

ED_Figure_1a - MIS-C CD3

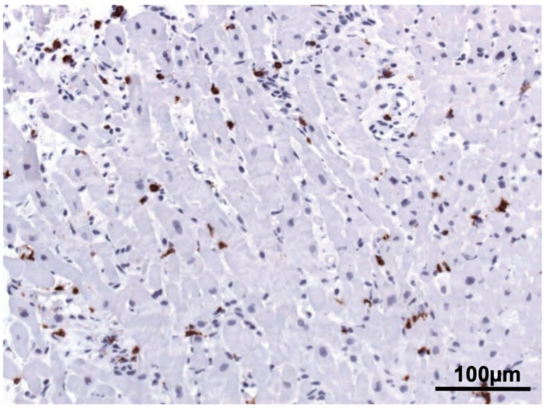

ED_Figure_1a - MIS-C CD68

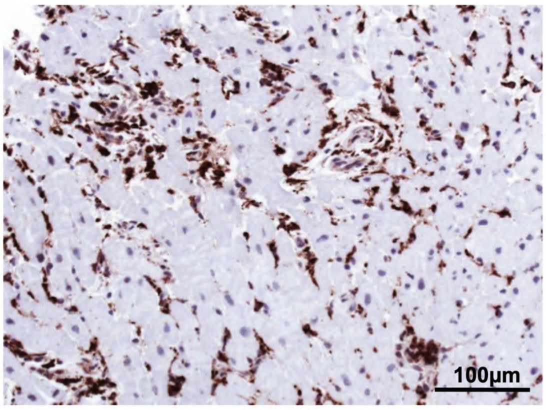

ED_Figure_1b - Non-COVID-19 CD4

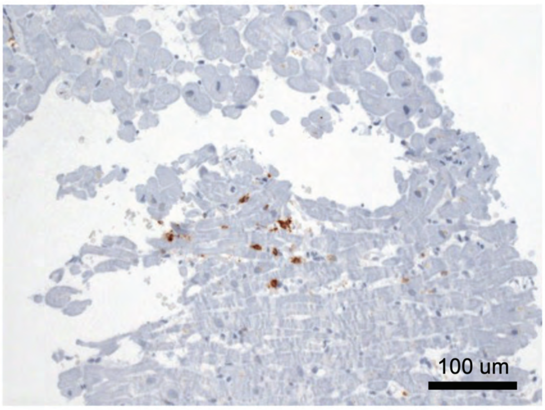

ED_Figure_1b - Non-COVID-19 CD8

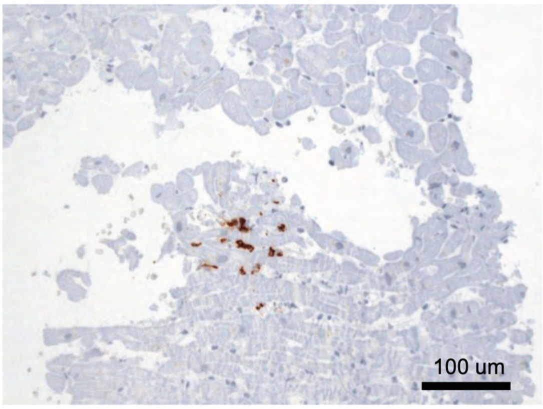

ED_Figure_1b - Post-COVID-19 CD4

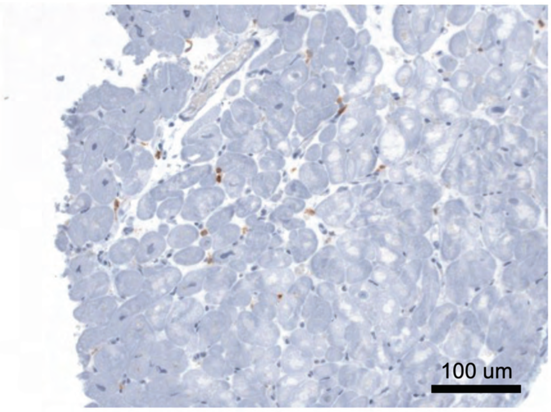

ED_Figure_1b - Post-COVID-19 CD8

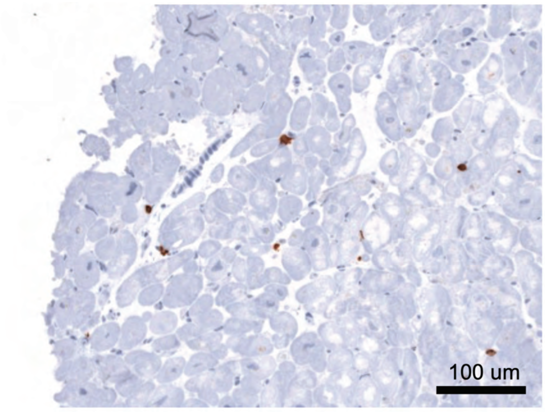

ED_Figure_1b - Post-Vaccination CD4

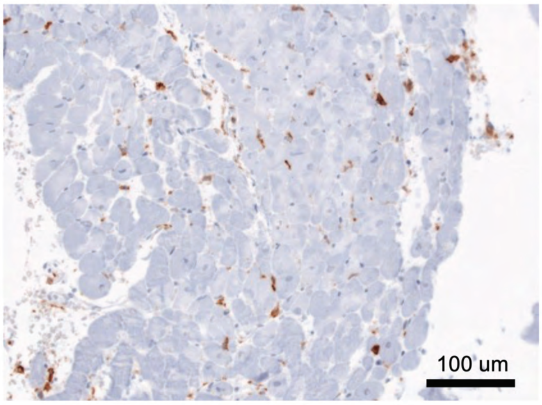

ED_Figure_1b - Post-Vaccination CD8

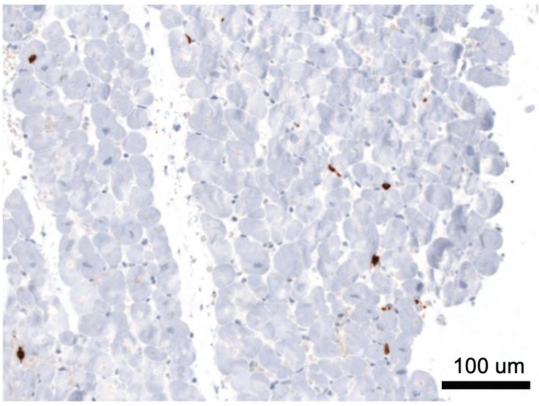

ED_Figure_1b - MIS-C CD4

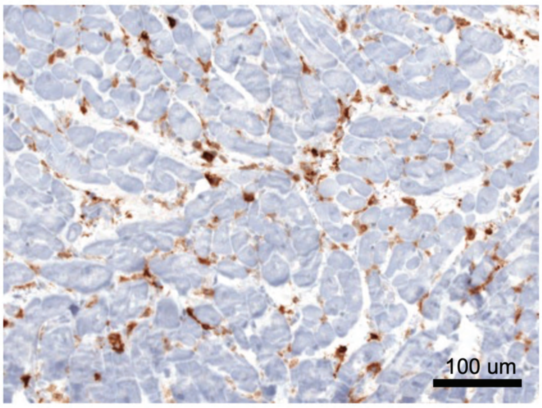

ED_Figure_1b - MIS-C CD8

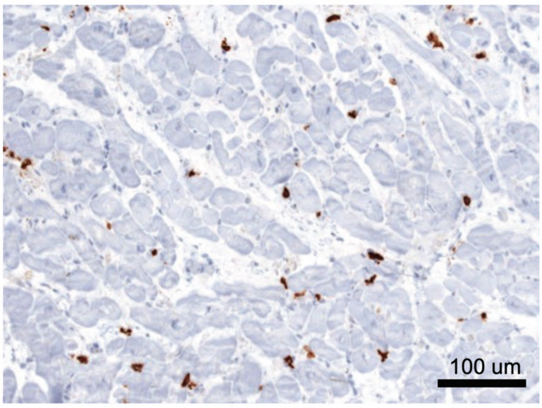

Supplement: Supplementary file 9 — Uncropped figures. [file 44161_2025_612_MOESM9_ESM.pdf]
